# Parametric investigation of ultrashort pulsed laser surface texturing on aluminium alloy 7075 for hydrophobicity enhancement

**DOI:** 10.1007/s00170-024-12971-8

**Published:** 2024-01-13

**Authors:** Abhijit Cholkar, Suman Chatterjee, Feljin Jose, Robert O’Connor, Éanna McCarthy, Nick Weston, David Kinahan, Dermot Brabazon

**Affiliations:** 1 I-Form, Advanced Manufacturing Research Centre, Dublin City University, Glasnevin, Dublin, Ireland; 2 Advanced Processing Technology Research Centre, School of Mechanical and Manufacturing Engineering, Dublin City University, Glasnevin, Dublin, Ireland; 3 DCU Water Institute, Dublin City University, Glasnevin, Dublin, Ireland; 4 School of Physical Sciences, Dublin City University, Glasnevin, Dublin, Ireland; 5 Renishaw Edinburgh, Riccarton, Edinburgh, EH14 4AP UK

**Keywords:** Hydrophobic surfaces, Ultrafast laser surface texturing, Laser process parameter optimization, Parametric modeling, Surface morphology, Surface chemistry

## Abstract

Hydrophobicity plays a pivotal role in mitigating surface fouling, corrosion, and icing in critical marine and aerospace environments. By employing ultrafast laser texturing, the characteristic properties of a material’s surface can be modified. This work investigates the potential of an advanced ultrafast laser texturing manufacturing process to enhance the hydrophobicity of aluminium alloy 7075. The surface properties were characterized using goniometry, 3D profilometry, SEM, and XPS analysis. The findings from this study show that the laser process parameters play a crucial role in the manufacturing of the required surface structures. Numerical optimization with response surface optimization was conducted to maximize the contact angle on these surfaces. The maximum water contact angle achieved was 142º, with an average height roughness (Sa) of 0.87 ± 0.075 µm, maximum height roughness (Sz) of 19.4 ± 2.12 µm, and texture aspect ratio of 0.042. This sample was manufactured with the process parameters of 3W laser power, 0.08 mm hatch distance, and a 3 mm/s scan speed. This study highlights the importance of laser process parameters in the manufacturing of the required surface structures and presents a parametric modeling approach that can be used to optimize the laser process parameters to obtain a specific surface morphology and hydrophobicity.

**Supplementary Information:**

The online version contains supplementary material available at 10.1007/s00170-024-12971-8.

## Introduction

Aluminium and aluminium alloys, especially the 6000 and 7000 series, are ubiquitous materials in the marine, aerospace, energy, medical, pharmaceutical, and food industries. This is because of their excellent physical and mechanical properties such as a high strength-to-weight ratio, strong corrosion resistance, and low cost [1]. In this wide range of applications, aluminium alloys come in contact with many fluids which contain foulants that can degrade the surface and other characteristic properties such as corrosion resistance, adhesion, and wear resistance over time [2–4]. To prevent or reduce this, surface modification of aluminium is required by developing a well-defined surface texture. This can be achieved using a technique that is non-toxic, cost-effective, robust, and environmentally friendly [5].

Surface topography and chemistry play an important role in various applications where the surfaces come into contact with different fluids. Enhanced surface functions can be achieved by controlling these surface properties, which in turn control the extent of the surface hydrophobicity or hydrophilicity. The surface topology can be modified using many existing chemical and physical-based techniques to obtain hydrophobic or even superhydrophobic surfaces on aluminium surfaces. Chemical etching [6], chemical vapor deposition [7], self-assembled monolayer formation [8], spray coating [9], dip coating [10], and sol–gel process [11] are some of the chemical-based techniques that are used traditionally. Physical texturing processes include lithography [12], electrospinning [13], plasma techniques [14], additive manufacturing [15], and electrochemical machining [16, 17]. However, laser-based nano and micro texturing methods have been used to produce textures on aluminium and aluminium alloys to change the wetting properties of their surfaces effectively. These laser-based manufacturing processes are capable of creating complex, precise, and consistent patterns with high dimensional resolution [18–20].

A number of studies have been conducted on the production of hydrophobic aluminium alloy surfaces through pulsed laser processing, as reported by various researchers including Ahuir-Torres et al. [19], Cardoso et al. [20], Milles et al. [21], Rukosuyev et al. [22], and Jagdheesh et al. [23]. The texture induced by pulsed laser on different surfaces offers advantages such as friction reduction between two surfaces, reduced drag, corrosion prevention by repelling water, as well as resistance to dirt and other particles, making them self-cleaning surfaces[24–27]. Consequently, such surfaces are easier to maintain and demonstrate increased resistance to fouling [28].

In recent years, the field of laser technology has experienced remarkable advancements, driven by extensive research and development endeavours. These efforts have led to notable breakthroughs, particularly in the domain of pulse durations. The continuous improvement in laser systems has successfully pushed the boundaries, resulting in pulse durations that now reach the femtosecond scale (10^–15^ s) [29]. Femtosecond (fs) laser surface texturing has numerous advantages such as the production of highly precise textures due to the reduced heat-affected zone compared to laser processing with higher pulse widths because of non-linear photon absorption, as observed by Valette et al. [30] and Malinauskas et al. [31]. The laser process parameters, including laser power [32], pulse repetition rate [33], scan speed, and hatch distance [34], can be adjusted to easily control the surface structures created by laser processing. The surface roughness and profile attributes of laser-structured surfaces can be tailored using femtosecond laser processing, as demonstrated in the early experiments conducted by and Martínez-Calderon et al. [35].

The achievement of hydrophobic and superhydrophobic properties on surfaces can be attributed to various factors, such as the low surface energy resulting from specialized tailored morphology, as reported by Tang et al. [36]. Surface textures consisting of repetitive patterns create an air boundary between the material surface, leading to low adhesion between fluid drops and the surface interface. Consequently, this alters the surface’s wetting properties, as discussed by Long et al. [37]. The wetting properties, especially on aluminium alloys, tend to change over time, and it is recommended to measure these properties once the surface textures have stabilized after environmental aging. For aluminium alloy, this stability is typically achieved between 45 and 60 days after laser processing. The duration may also depend on the complexity of the surface structures, as noted by Milles [28]. Contact angle measurements are commonly used to assess surface wettability, which involves the dispensing of a water droplet onto the surface and measuring the angles between the solid–liquid-gas interfaces [38]. Over the past years, several researchers have worked on understanding the effect of different laser process parameters separately [39–42]; however, the investigation of the correlation of the laser process parameters and surface properties such as surface roughness and wettability especially on aluminium alloy 7075 requires further research to provide sufficient fundamental understanding to create tailored surfaces and identify the best laser processing settings [43, 44]. To understand the effect of the input laser parameters and determine the correlation with the wettability process responses such as contact angle, a design of experiment layout is often used to relate the process inputs and output responses [45]. In the past literature, a lot of research has been conducted on the modification of morphology using different types of laser systems on different materials for various applications; however, the area effect of process parameters in femtosecond laser processing to develop highly hydrophobic surfaces on aluminium remains largely unexplored.

In this research, an experimental study of the manufacturing of surface textures on an aluminium alloy (7075) surface employing an advanced femtosecond laser surface texturing process using the design of experiments approach is presented. Surface height roughness (Sz) and spatial roughness (Sa) profile parameters, surface profile aspect ratio (Str), and contact angle were measured as output responses. The influential factors such as laser power, hatch distance, and scan speed of the femtosecond laser system are examined in this paper. The process parameters resulting in the most hydrophobic surface were determined using response surface optimization. Relatively little is known about how the different femtosecond laser process parameters affect the surface roughness and chemical composition, and surface wettability. The findings underscore the importance of the interplay between laser power, hatch distance, and scan speed in achieving optimal hydrophobicity. This represents a noteworthy advancement, as it not only establishes a correlation between process parameters and surface properties but also elucidates the significance of these parameters in determining the effectiveness of the manufactured surfaces in resisting water contact. This study was targeted to fill this gap of knowledge and develop an understanding of the main reasons and phenomena behind these effects which can further enhance the development of antifouling and corrosion resistance surfaces, especially for marine applications.

## Materials and methods

### Materials

Aluminium alloy 7075-T651 with the chemical composition of 0.1% Si, 0.22% Fe, 1.5% Cu, 0.17% Mn, 2.8% Mg, 0.21%, 5.8% Zn, 0.03% Ti, and Al Balance, supplied by Impact Ireland Metals Ltd and was thermally treated to stress-relieve by stretching and artificially aged. The primary alloying element in this aluminium alloy is zinc which makes the material highly ductile, with excellent mechanical properties such as a yield strength of 507 MPa, ultimate tensile strength of 574 MPa, and hardness of 171 HB. The surface roughness of the as-received samples was Sa = 492 nm as measured using a Bruker Contour GT-X Profilometer. The test samples were sliced to the dimensions of 80 × 80 mm with a thickness of 2 mm. The surface of the samples was cleaned using isopropyl alcohol before laser processing to clean away any dust particles or other contaminants. The spectral absorbance of the laser light at 1030 nm wavelength (the laser wavelength used in this study) for aluminium alloy 7075 is 0.7 at room temperature as reported by El-Hameed et al. [46].

### Experimental process

**Fig. 1:**
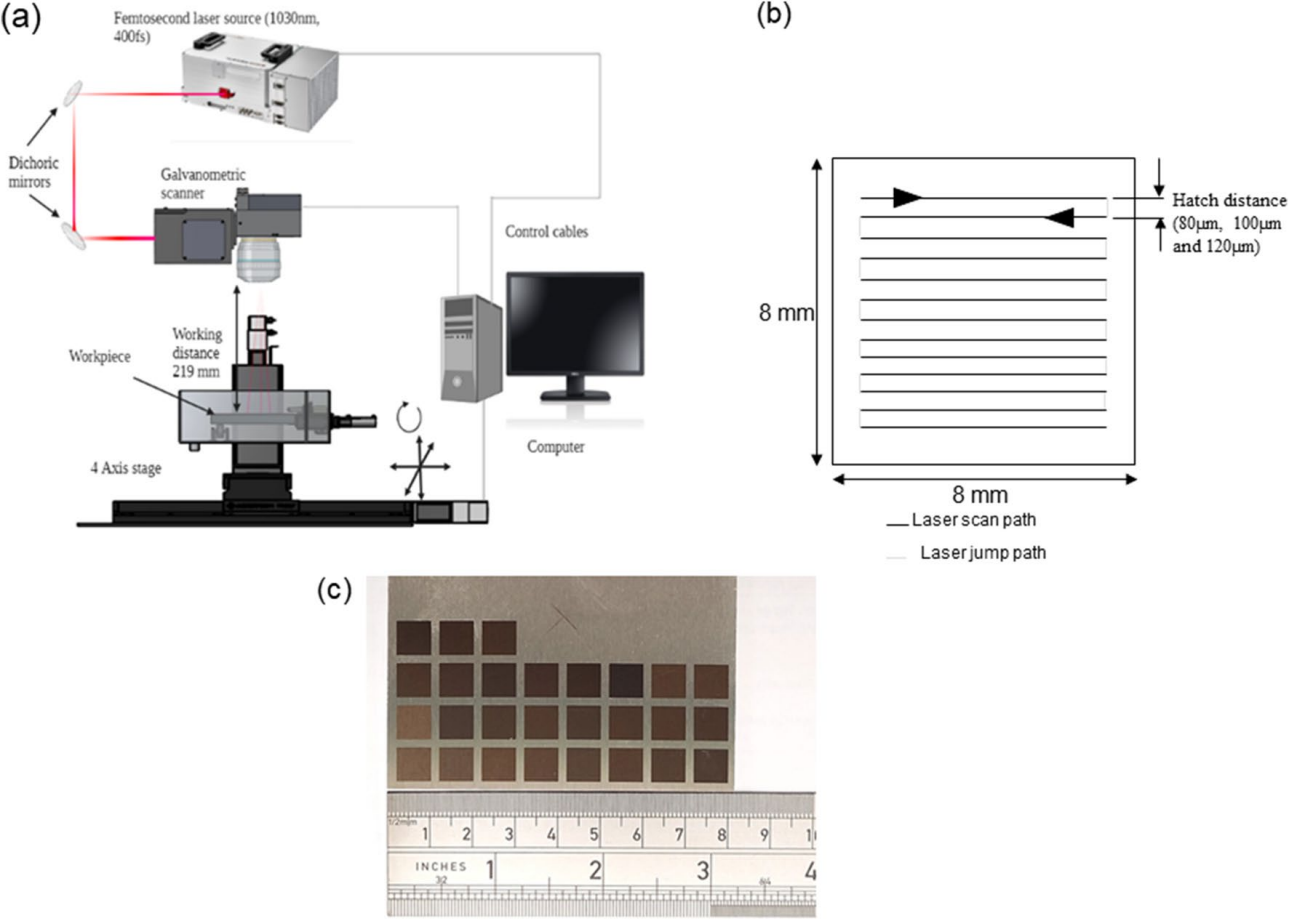
Schematics of (**a**) laser surface texturing setup and (**b**) the laser scanning raster pattern. (**c**) Actual sample image after laser treatment

The laser surface texturing was conducted using a high-energy industrial ultrafast femtosecond laser source (NKT Origami XP). This laser provides a stable, low noise, pulsed beam with an average wavelength of 1030 nm, a pulse duration < 370 fs, average power of 4 W, and a maximum repetition rate of 1 MHz. An integrated galvanometer beam scanner (FOCUSSHIFTER-15, RAYLASE GmbH) was used to control the scanning of the laser beam over the sample surface. The beam was focused on the sample surface using an F-theta lens with a working distance of 219 mm from the lens. The experimental setup and scan pattern used are shown in Fig. 1(a).

The beam has a Gaussian profile with a diameter (1/e^2^) of 51.2 µm at the focal position, as measured with a CMOS camera beam profiler (detailed in supplementary information).

The samples were mounted and fixed on four axes positioning stage 225P from Aerotech Inc, which was used to set the laser beam focus at the sample surface. A bidirectional scanning strategy was employed for manufacturing surface textures as shown in Fig. 1(b). The surface texturing was conducted over areas of 8 mm × 8 mm at different positions on the sample with different process parameter settings. The laser processing was conducted in the atmospheric environment at a room temperature of 20º C and humidity of 75%. The samples were cleaned with isopropyl alcohol after laser texturing to remove the dust particles and then stored in an airtight petri dish in a clean environment for aging for 60 days.

### Experimental design

A full factorial Design of Experiment (DoE) model of 3^3^ (three factors at three levels) was developed in order to investigate the effect of the input processing parameters on the output characteristics. The input parameters were the laser power, the scanning speed, and the hatch distance, see Table 1. A preliminary test was conducted and carried out to identify the most significant processing parameters and their level of significance for process optimisation.

**Table 1:** The design of experiments (L27) laser process parameters and levels examined

Parameter	Unit	Level 1	Level 2	Level 3
A Laser power (P)	W	3	3.5	4
B Hatch distance (*h*)	mm	0.08	0.1	0.12
C Scan speed (*v*)	mm/s	3	4	5

The pulse repetition rate was fixed at 100 kHz. The laser power was varied between 3, 3.5, and 4 W, the scan speed was varied between 3, 4, and 5 mm/s and the hatch distance were varied between 0.08 mm, 0.1 mm, and 0.12 µm. The scanning pattern was raster scanned parallel lines (using a bidirectional scan strategy) with the noted process parameters. Laser texturing is contingent upon the attainment of a specific fluence of the laser that surpasses the ablation threshold value inherent to the material under consideration. During the preliminary experimental phase, optimal textural outcomes were observed when the laser power was configured to exceed 3 W. Given the maximum power setting of 4 W in the employed laser system, power levels within the range of 3 to 4 W were systematically investigated. The hatch distance range was selected based on the diameter of the laser beam spot, as this parameter has a direct impact on the resolution and quality of the resulting processed features. Similarly, the scan speed was selected based on pilot tests conducted with the laser setup and material. Table 2 shows the experimental parametric settings for each of the 27 laser-fabricated samples. Each sample was manufactured and tested three times in order to examine the repeatability of the results. Therefore, 81 samples were manufactured and characterized.

**Table 2:** Full listing of the process parameters and associated levels investigated for each sample

Sample	Laser power (W)	Hatch distance (mm)	Scan speed (mm/s)
1	3	0.08	3
2	3	0.08	4
3	3	0.08	5
4	3	0.1	3
5	3	0.1	4
6	3	0.1	5
7	3	0.12	3
8	3	0.12	4
9	3	0.12	5
10	3.5	0.08	3
11	3.5	0.08	4
12	3.5	0.08	5
13	3.5	0.1	3
14	3.5	0.1	4
15	3.5	0.1	5
16	3.5	0.12	3
17	3.5	0.12	4
18	3.5	0.12	5
19	4	0.08	3
20	4	0.08	4
21	4	0.08	5
22	4	0.1	3
23	4	0.1	4
24	4	0.1	5
25	4	0.12	3
26	4	0.12	4
27	4	0.12	5

Response surface method (RSM) of experimental design is a set of mathematical and statistical techniques that is useful for modeling and predicting the output response. This was conducted to explore the relationship between the input laser process parameter factors and the responses of Sa, Sz, and Str, as defined in the introduction section, the surface composition, and the water contact angle. The response data obtained from the experiments underwent analysis using Design-Expert 13 and Minitab 18 software. Only models with high significance, and no significant lack of fit in the data, are presented in the results section. The surface response equations expressed the actual factors and levels of process parameters and included coefficients accounting for the actual units of each parameter.

### Surface characterization

#### Surface morphology

The laser-treated surfaces were evaluated using scanning electron microscopy (SEM), EVO LS15 (Zeiss) with LaB6 filament, an accelerating voltage of 10 kV, and a beam current intensity of 25 pA.

#### Surface roughness

The different surface roughness attributes such as arithmetical mean height (S_a_), maximum height (S_z_), and aspect ratio (S_tr_) were investigated as areal parameters of the surface roughness. S_tr_, surface texture aspect ratio, is defined as the ratio of the average width of the surface features to the average height of the surface features. These are considered in this study as per ISO 25178–2 (2021) [47]. These were characterized using a 3D Bruker Contour GT-X Profilometer (Billerica, MA, USA). The measurements were made using a 5X lens focusing on a 1-mm^2^ surface area. The acquired data was analysed using Bruker Vision 64® software. The results were plotted considering the average and the standard deviation calculated on the basis of the three experiments carried out for each set of processing conditions.

#### Wettability

Prior to the wettability test, the samples were carefully prepared by drying and cleaning using compressed air to remove any debris or contaminants. The static water contact angle measurements were performed using a FTA200 Dynamic Contact Angle Analyser. The sessile drop profile was measured using the non-spherical liquid–vapor curve fit method within the FTA 200 software. These measurements were conducted at ambient conditions (16 °C and 86% relative humidity). For the measurements, approximately 10–18 µl volume of deionized water was used for each measurement. The average static contact angle is reported in this study with (*n* = 3). The experiment was performed on three separate samples, with each instance using the same model to ensure reproducibility. The contact angle test was conducted on the samples on day 1 of the laser texturing and after 60 days of environmental aging as the surface textures were thermodynamically stabilized.

#### Surface elemental composition

The surface chemistry of the irradiated materials was analysed by X- Ray photoelectron spectroscopy (XPS). In this study, Scienta Omicron XPS system equipped with a monochromatic Al Kα X-ray source (operated at 240W and 8 kV) at base pressure setup at 6 × 10^–7^ Pa with a 128 channel Argus CU detector was employed. The kinetic energy of the photoelectrons was determined using an analyser with a pass energy of 100 eV to survey and 50 eV for high-resolution spectra. The take-off angle between sample’s surface normal and the electron optical axis of the spectrometer was 0°. Spectra were referenced to the C1s peak of aliphatic carbon at a binding energy of 284.8 eV. CasaXPS software (Casa Software Ltd., UK) was used to analyse the results using satellite subtraction procedure. Quantitative elemental compositions were determined from peak areas using this method.

## Results

### Design of experiment results

In this experimental design, the effects of laser power, hatch distance, and scan speed on surface roughness and contact angle were investigated. The response variables were measured using a 3D profilometer and a goniometer, respectively. The initial samples were not subjected to any mechanical or chemical treatment and had an average surface roughness Sa of 0.5 ± 0.005 µm, maximum height roughness of 6.82 ± 3.36 µm, and a texture aspect ratio of 0.06 ± 0.01. To ensure reproducibility and repeatability, measurements were conducted on three different samples with the same process parameters. Table 3 shows the resulting average surface profile values measured for surface roughness attributes Sa, Sz, Str, and contact angle. Samples 1 and 22 (green highlight) show the highest contact angles recorded, and samples 12 and 17 (orange highlight) show the lowest recorded contact angle measurements. These samples were analysed using SEM and XPS.

**Table 3:**
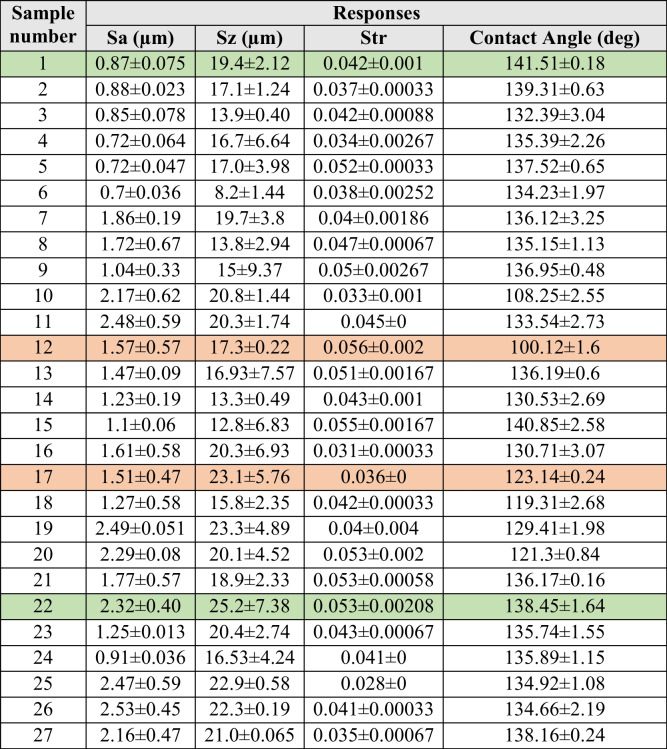
Resulting average surface profile values measured for surface roughness attributes Sa, Sz, Str, and contact angles

#### Input–output correlation

To investigate potential correlations between input process parameters and the corresponding responses, Pearson correlation coefficients were employed. To gain insights into the strength and direction of correlations between multiple variables, we utilized a heat map of Pearson correlations coefficient as shown in Table 4. The heat map shows the correlation of the input process parameters and the response variables. The analysis of the correlation and the scatter plots are provided in the supplementary document. A negative r value indicates an inverse relation.

**Table 4:**
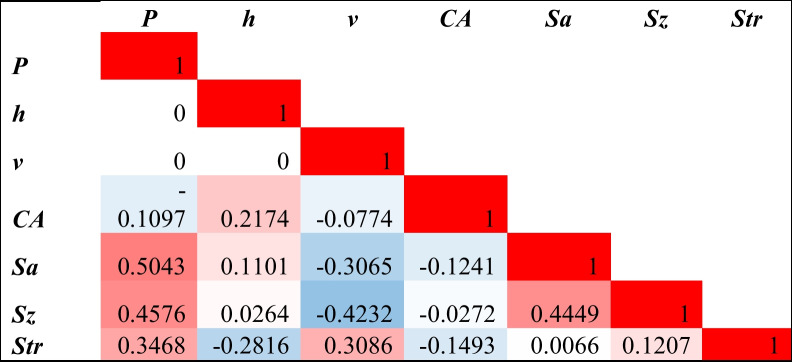
Pearson correlation heat map of input parameters (laser power, hatch distance, and scan speed) and output responses (contact angle, Sa, Sz, and Str)

#### Significance analysis

Design Expert 13 DOE software was employed to analyse the output data and develop models that describe the relationship between the processing parameters and the resulting responses. The ANOVA tables and details are provided in the supplementary information. Four statistically significant model equations were derived from the recorded responses, which describe the effects on the contact angle (a measure of wetting behaviour), surface mean height, surface maximum height (based on the difference between the height of peaks and depth of valleys across the surface area), and the texture aspect ratio (which relates to the uniformity of the surface texture). The results of the ANOVA analyses for these models are presented in Table 5.

**Table 5:** Goodness of fit statistical measures for the developed models

Response	Degree of freedom	R^2^	Adequate precision
Arithmetic mean height (Sa)	12	0.80	15.29
Maximum height (Sz)	9	0.75	9.5
Texture aspect ratio (Str)	16	0.87	14.36
Contact angle (CA)	20	0.91	25.22

The R^2^ values for all the models were greater than 0.75 which shows that the models were significant. Moreover, it can be seen from this table that adequate precision for all the models is greater than 9. If the ratio surpasses 4, the model equations are regarded as having attained a satisfactory level of precision [48, 49].

#### Effect of laser surface texturing on height roughness parameters (Sa, Sz)

**Fig. 2:**
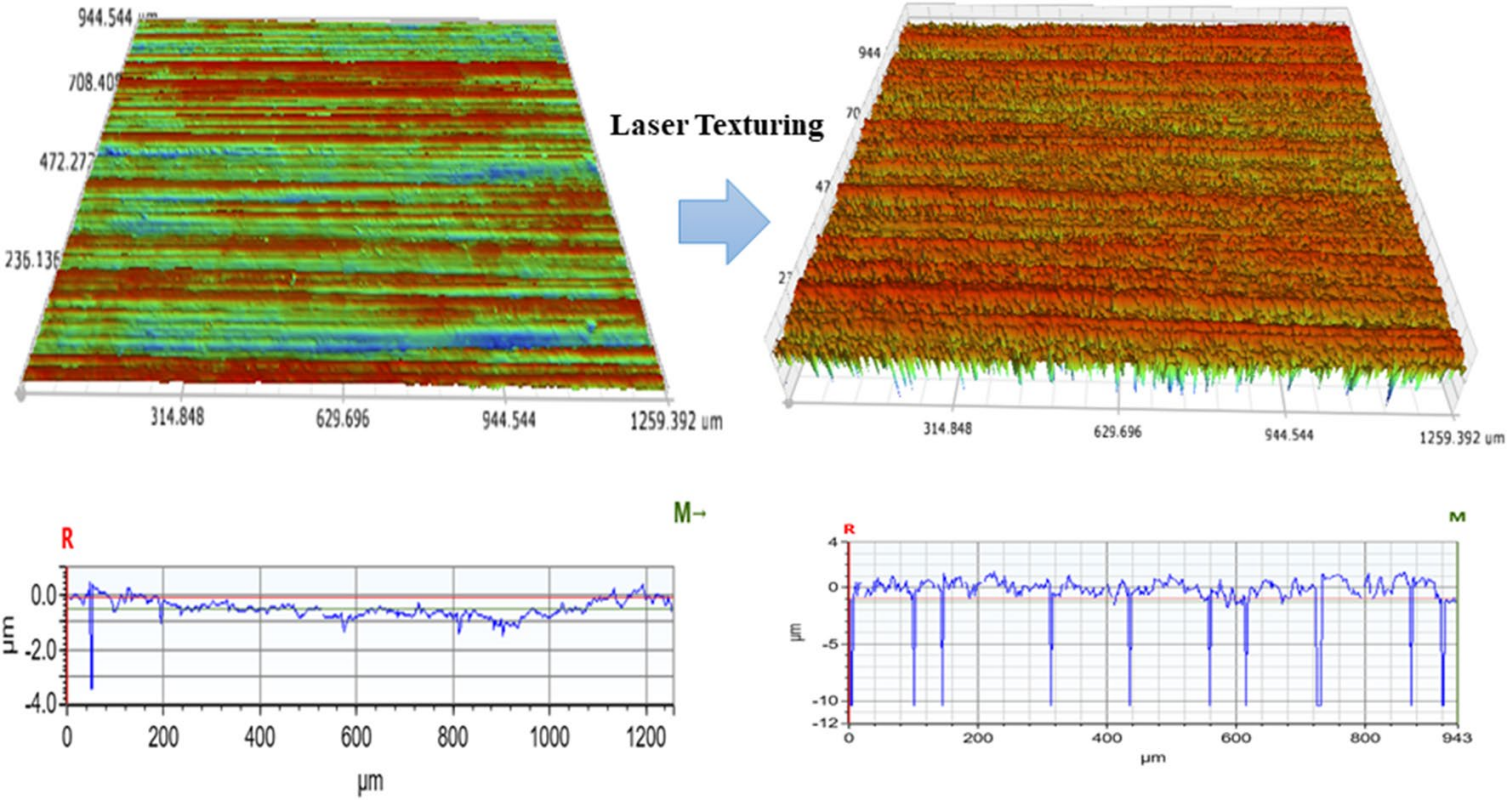
Change in surface morphology after laser surface texturing as observed on the 3D optical profilometer of sample 1

The surface roughness measurements were done using 3D optical profilometer that uses white light interferometry and measures the roughness height attributes as per ISO 25178–2 (2008) [47]. Figure 2 shows the surface morphology change after laser texturing, as observed on 3D profilometer.

**Fig. 3:**
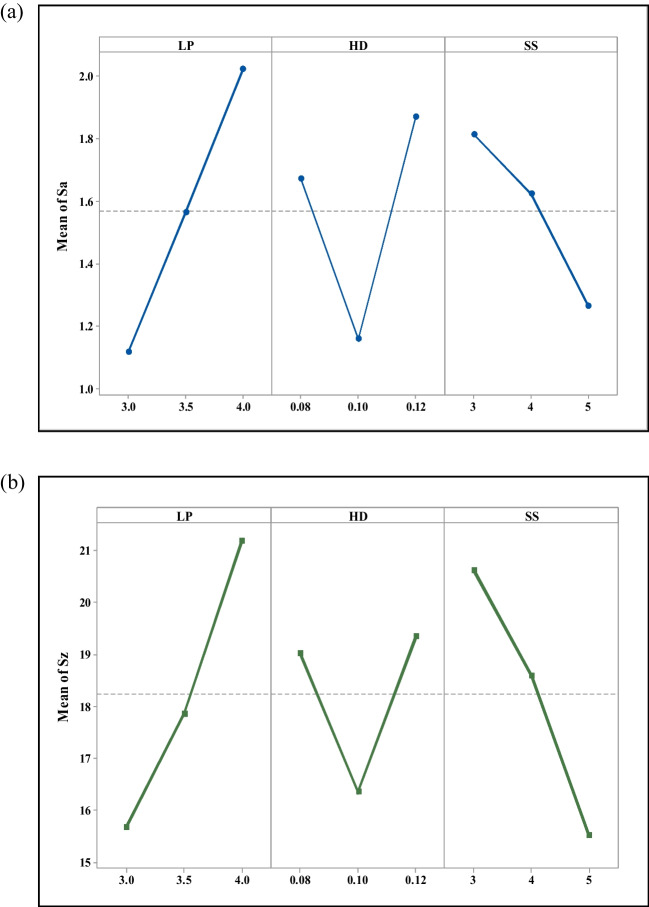
Main effect plots for (**a**) the arithmetic mean surface roughness (Sa) and (**b**) the maximum height roughness (Sz); showing the effects of the input processing parameters of LP, laser power; HD, hatch distance; and SS, scan speed

**Fig. 4:**
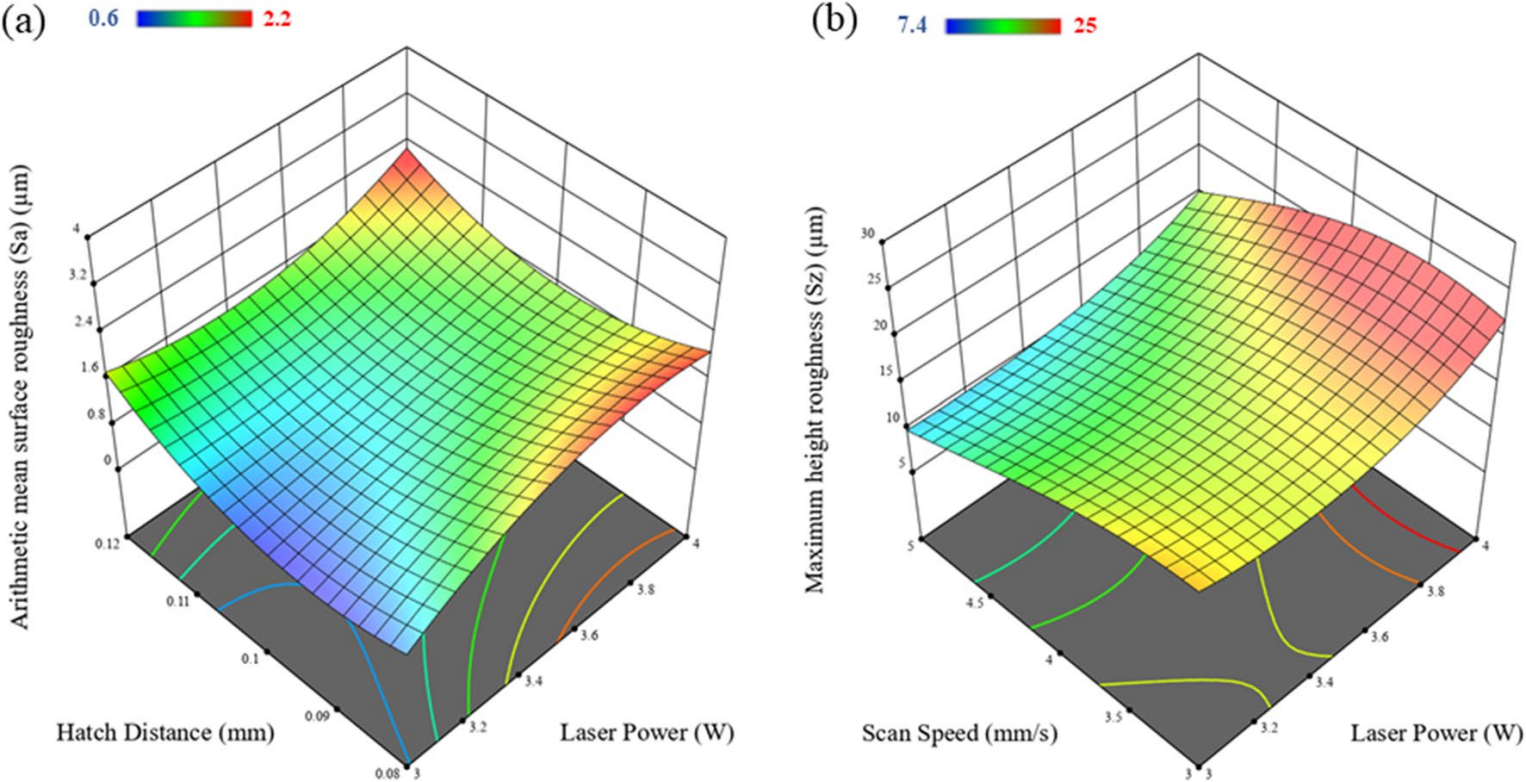
Response graphs showing the effect of the input processing parameters on the output measured surface roughness (**a**) arithmetic mean surface roughness (Sa) and (**b**) the maximum height roughness (Sz)

ANOVA analysis through the DoE softwares Minitab and Design Expert was conducted in which two significant mathematical models were discovered. These models indicate the features associated with the surface height roughness of the samples with respect to the laser process parameters selected. The regression equation derived for Sa and Sz were as follows.

1 $$\mathbf{S}\mathbf{a}=-38.5008 + 12.6472\mathrm{ P }+ 336.749\mathrm{ h }+ 8.9592\mathrm{ v}-131.962\mathrm{ Ph}-1.8307\mathrm{ Pv}-87.0903\mathrm{ hv }+ 561.985 {{\text{h}}}^{2} + 9.7049\mathrm{ Phv}+ 2.93506 {{\text{P}}}^{2}\mathrm{h }+ 69.1836 {{\text{Ph}}}^{2}{\text{v}}$$

2 $$\mathbf{S}\mathbf{z}\boldsymbol{ }\boldsymbol{ }= -4035.03 + 2442.32\mathrm{ P }+ 83961.4\mathrm{ h}-5.7796\mathrm{ v}-50444.4\mathrm{ Ph }+ 0.6444\mathrm{ Pv }+ 9.7222\mathrm{ hv}-355.4 {{\text{P}}}^{2}-420347{{\text{h}}}^{2} + 7341.11{{\text{P}}}^{2}\mathrm{h }+ 252194 {{\text{Ph}}}^{2}-36666.7 * {{\text{P}}}^{2}{{\text{h}}}^{2}$$

where *P* is the laser power measured in W, *h* is the hatching distance measured in mm, and *v* is the laser scan speed measured in mm/s. The mean effect plots in Fig. 3 and response surface graphs in Fig. 4 were generated by applying the model Eqs. (1) and (2) for surface roughness parameters Sa and Sz, as shown in the above section.

Laser power was identified as the most influential parameter on the height roughness parameters, with a direct relationship to the laser energy density. These findings are consistent with the results previously reported by Zang et al. [38]. Laser scan speed was found to be the second most influential parameter and Sa values shows a declining trend with increase in scan speed. However, changes in hatch distance were found to greatly impact the mean roughness (Sa) of the surface. An increasing trend in arithmetic mean roughness (Sa) was observed with increasing laser power. The mean of Sa decreased with an increase in hatch distance from 0.08 to 0.1 mm, but the trend changed drastically between 0.1 and 0.12 mm. An increasing trend was observed. A similar trend of mean of Sa was observed in the literature by Wang et al. [39]. The Sa value was observed to rise with the increase in laser power from 3 to 4 W, as shown in Fig. 3(a). Furthermore, the hatch distance was determined to be another crucial factor in getting higher Sa values at hatch distances of 0.08- and 0.12 mm, while lower Sa values were observed for hatch distances close to 0.1 mm. Figure 3(b) showed that there is a linear correlation between increasing laser power and Sz values, and the Sz values indicated a decrease in Sz with an increase in laser scan speed. The agreement of this model with the experimental results was evaluated with the aid of a normal plot of the residuals and a plot of the predicted vs actual values (see supplementary information). It can be seen from these graphs that deviations from the model are approximately normal and there are no significant outliers in either dataset.

#### Effect of laser surface texturing on spatial roughness parameters

Texture uniformity is an important response parameter in laser surface texturing because it is a measure of how consistent the surface structures are across the entire surface. A uniform surface texture will have a consistent pattern of peaks and valleys, with similar shapes and sizes, resulting in a more reliable and consistent behaviour when the surface comes into contact with different fluids. The regression equation for Str is as follows:

3 $$\mathbf{S}\mathbf{t}\mathbf{r}= 6.4377 -3.8912 P -25.568 h + 4.7648 v + 1.1947Ph-67.0004 hv + 0.85492 {P}^{2} -0.6534 {v}^{2} -0.4969{P}^{2}v-31.9142P{h}^{2} +0.2717P{v}^{2} + 194.46 {h}^{2}v + 6.2146 {P}^{2}hv-0.0085 {P}^{2}{v}^{2} + 105.808{Ph}^{2}v-0.5173 Ph{v}^{2} -42.4881{P}^{2}{h}^{2}v$$

where* P* is the laser power measured in W, *h* is hatching distance measured in mm and *v* is the laser scan speed measured in mm/s.

**Fig. 5:**
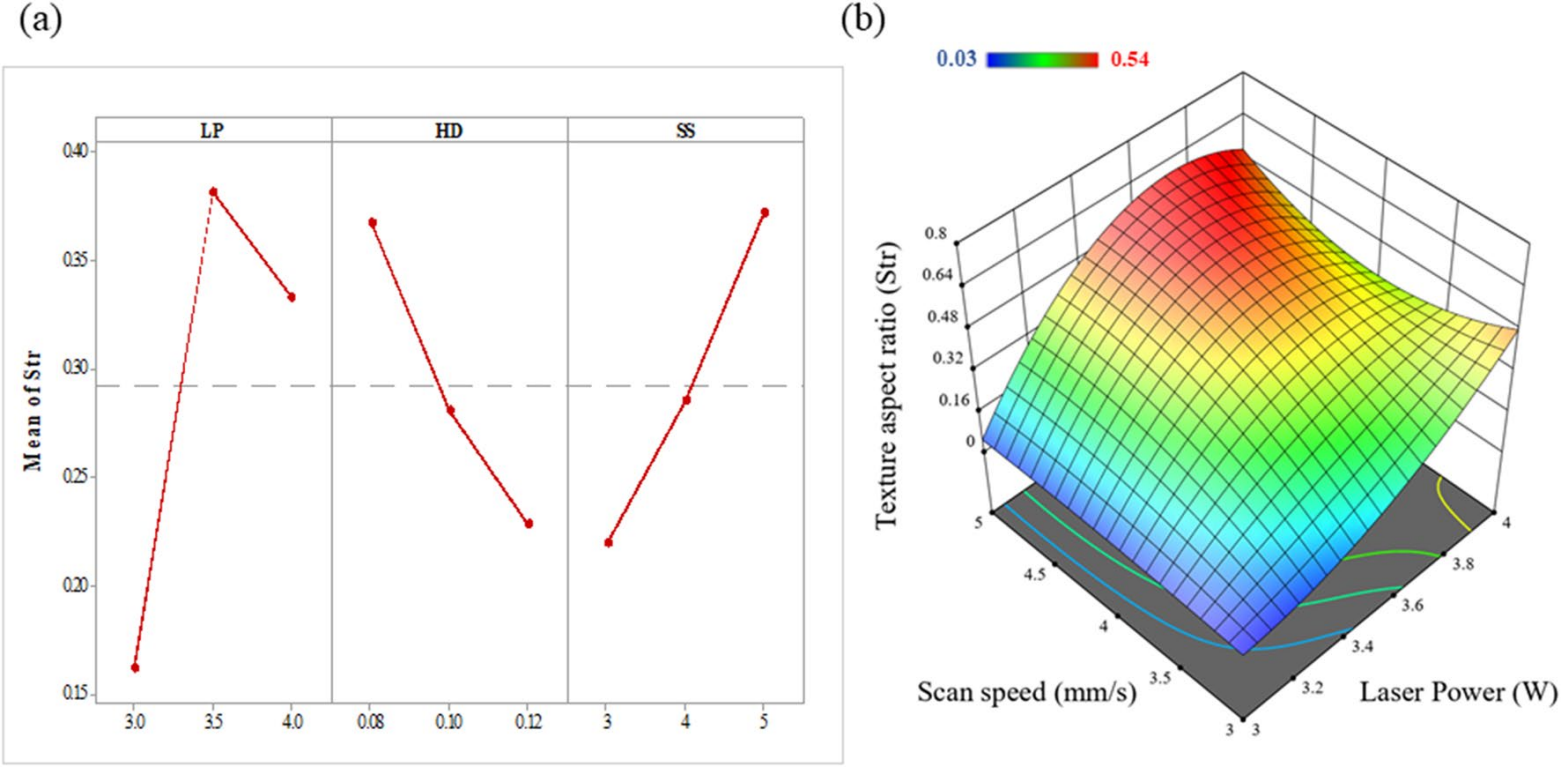
(**a**) Main effect plot for texture aspect ratio surface roughness (Str) showing the effect of the input processing parameter such as LP- laser power, HD- hatch distance and SS- scan speed, and (**b**) 3D response surface plots for Str

The mean effect plot and response surface 3d plot is illustrated in Fig. 5. These plots were generated by applying the model Eq. (3) for surface texture aspect ratio (Str).

As the size of the laser spot is smaller than the step-over distance, the laser will only affect discrete areas of the material during each pass, resulting in a pattern of parallel lines with un-affected gaps between them. This observation is consistent with the principles of laser-material interaction, where the size and shape of the laser spot and the scan path determine the spatial distribution and extent of the laser affected area on the surface. From the mean effect plots illustrated in Fig. 5(a), laser power and laser scan speed are the significant laser process parameters affecting the texture aspect ratio. When the laser power is increased, more energy is delivered to the material surface, causing more melting and evaporation, and resulting in a greater degree of surface roughness. This leads to an increase in the height component of the surface roughness, which results in an increase in the texture aspect ratio. The linear increasing relationship can be seen in the mean of Str with increasing scan speed.

This trend can be observed in the 3D response surface plot shown in Fig. 5(b). Higher Str value is seen when the laser power is 4 W and scan speed is 5 mm/s, and lower Str value is shown at 3 W power and 3 mm/s.

The model’s compatibility with experimental results was assessed using a normal plot of residuals and a predicted vs. actual values plot (refer to supplementary information). The analysis indicated that any deviations from the model followed an approximately normal distribution, and there were no noteworthy outliers observed in the data.

#### Effect of laser surface texturing on wettability

The contact angle serves as a quantitative assessment of the surface wetting characteristics, whereby a higher contact angle signifies a hydrophobic surface that repels water, while a lower contact angle indicates a hydrophilic surface that attracts water. In the instance of the non-treated aluminium alloy surface, Fig. 4 demonstrates a contact angle measurement of 84.91º. This measured contact angle signifies the hydrophilic nature of the surface, implying its high propensity to be wetted by water.

**Fig. 6:**
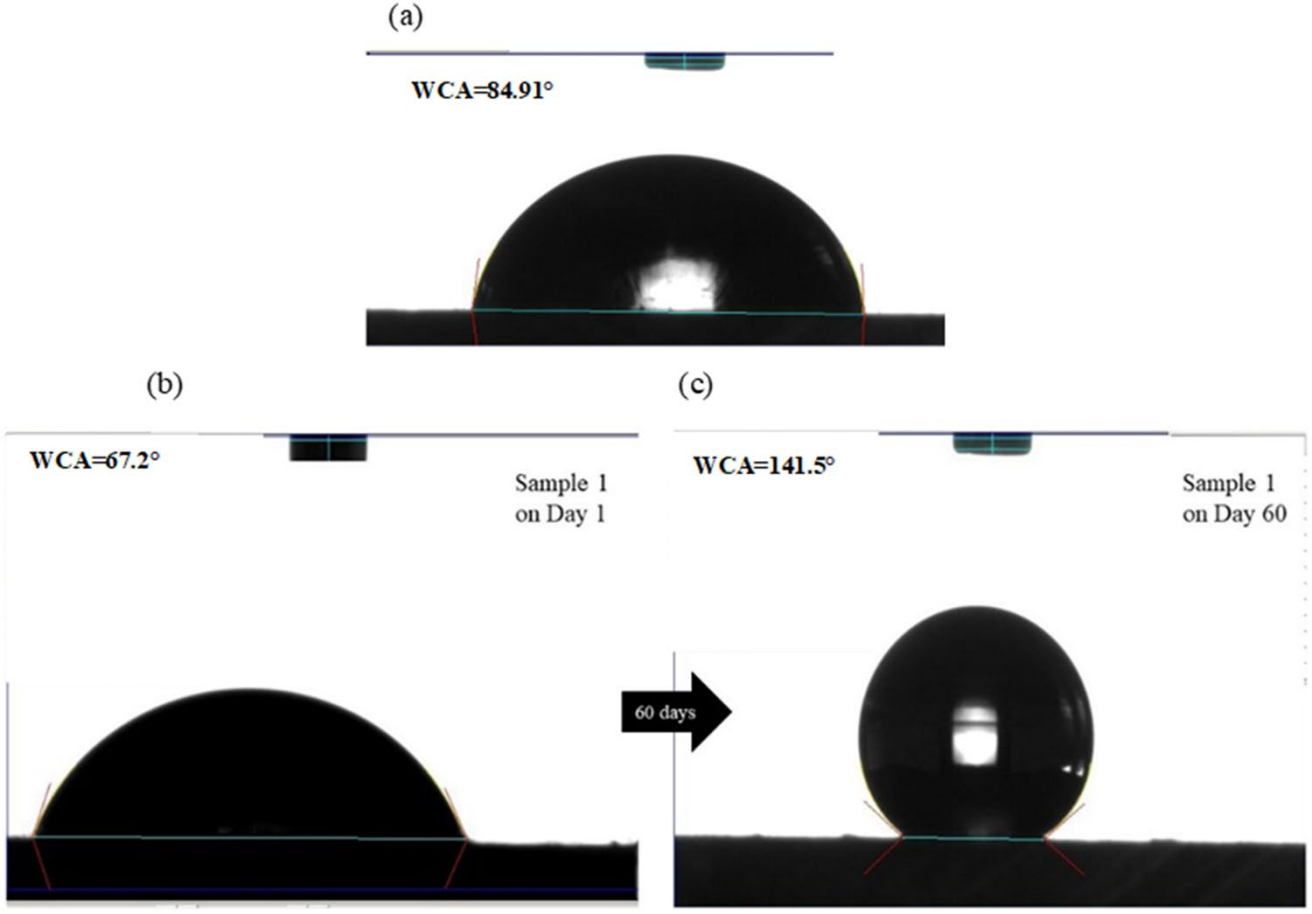
(**a**) Picture of a water droplet on the non-structured aluminium 7075 sample with contact angle of 84.9°; (**b**) a water droplet on laser structured aluminium on day 1 showing hydrophilicity with a water contact angle 67.2° and (**c**) a water droplet on laser structured aluminium surface after 60 days showing hydrophobicity with a contact angle of 141.5°

Prior to the aging process, the contact angles observed on laser-textured specimens (shown in Table 3) fell within the range of 11–65°. All of these values represent a substantially more hydrophilic surface than the non-structured sample, which displayed a contact angle of 84.91°. However, within a 60-day timeframe, the contact angles of the laser-textured samples progressively increased, reaching the range of 100–142°, as illustrated in Fig. 6 for the same sample (sample 1). This suggests that the initial instability of the laser-induced surface structures is overcome through an aging process, potentially involving chemical reactions such as oxidation and/or diffusion triggered by environmental exposure. The attainment of surface wettability stability has been previously reported by several researchers after a duration of 45 days [43–45]. Thus, in this study, the contact angle measurements were taken after 60 days. The results demonstrate that the wettability of the laser structured aluminium alloy is significantly affected by the laser process parameters such as laser power, hatch distance, and scan speed, as well as aging time. The regression equation derived for contact angle is as follows:

4 $$\mathbf{C}\mathbf{o}\mathbf{n}\mathbf{t}\mathbf{a}\mathbf{c}\mathbf{t}\mathbf{A}\mathbf{n}\mathbf{g}\mathbf{l}{\text{e}}= 7.92496 -23768.8 h +376.878 v +7126.99 Ph-6.6317 {P}^{2} + 200042 {h}^{2} +96.6219 {v}^{2} +123.041Phv-42.5001 {P}^{2}h-24.8459 {P}^{2}v-53516.7 {Ph}^{2}-102.228 {Pv}^{2}-4515.88 {h}^{2}v-1269.25 {hv}^{2} +19.871 {P}^{2}{v}^{2}-14494.1 {Ph}^{2}v +1078.7 {Phv}^{2} +776.929 {h}^{2}{v}^{2} +3626.42{P}^{2}{h}^{2}-196.751 {P}^{2}{hv}^{2}-6.1761 {P}^{2}{h}^{2}{v}^{2}$$

where* P* is the laser power measured in W, *h* is hatching distance measured in mm, and *v* is the laser scan speed measured in mm/s.

**Fig. 7:**
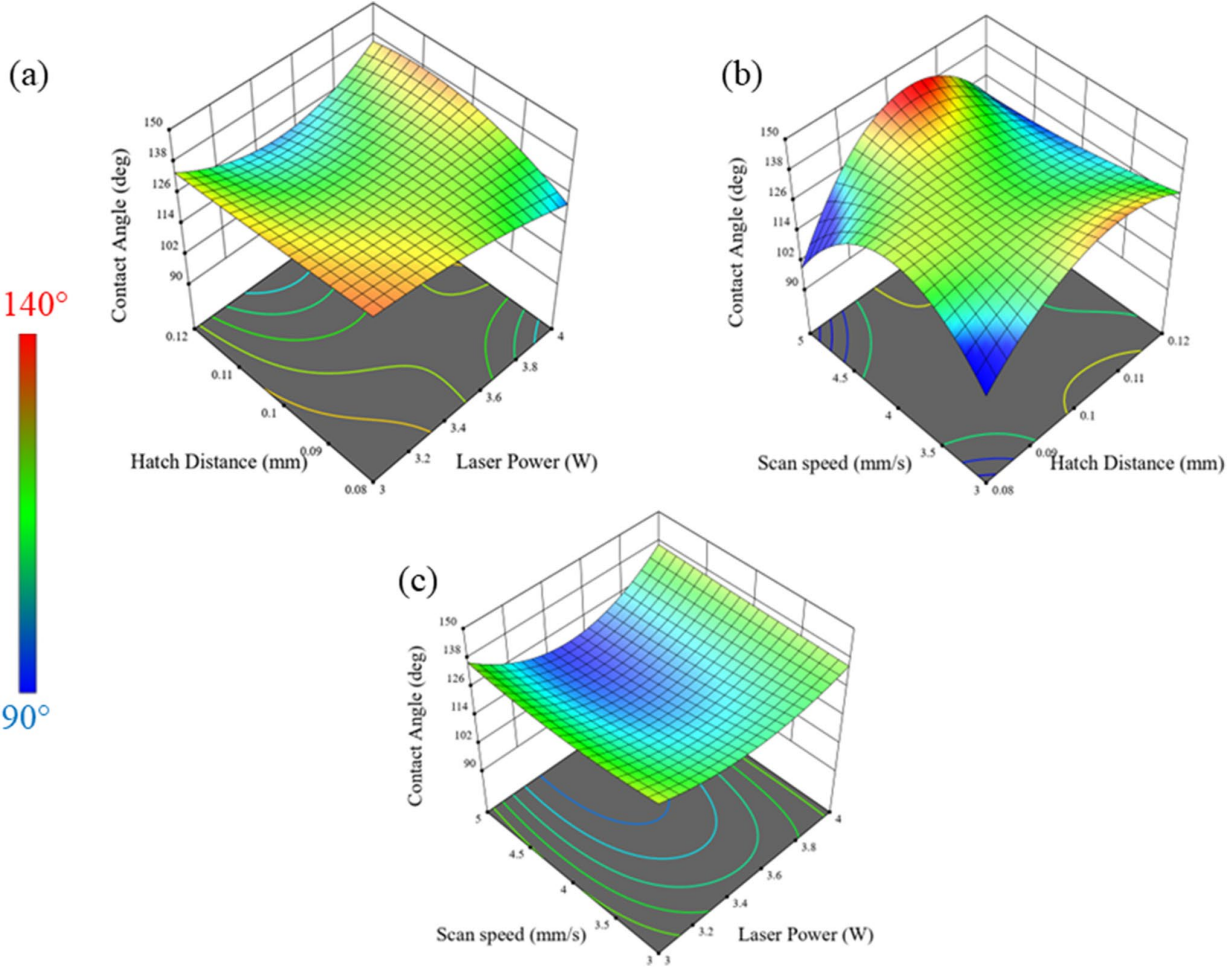
Response surface plots and contour plots of the contact angle shown from derived model at constant (**a**) scan speed (*v* = 4 mm/s), (**b**) laser power (*P* = 3.5 W*)*, and (**c**) hatch distance (*h* = 0.1 mm*)*

Figure 7 shows the response surface graphs showing change in water contact angle with respect to change in laser power set at 3 W, 3.5 W, and 4 W. The graphs suggests that when the laser power is set at 3 W, the highest contact angle will be achieved at 0.8 mm hatch and 3 mm/s. However, when laser power is set at 3.5 W, higher more hydrophobic surfaces can be achieved with hatch distance 0.1 mm and at higher speed of 5 mm/s.

When the laser power is set to 4 W, more hydrophobicity can be achieved when the hatch distance is set to be greater than 100 μm, and there is not much effect of the scan speed on the trend. This indicates that the surface structures are created efficiently when there is interaction between the laser and the material, and there is enough fluence to structure the surface of the material.

#### Numerical optimization

The numerical response optimization was also conducted in Design expert 13 software in order to find out the optimal laser surface texturing conditions at which the desirable surface properties such as increased hydrophobicity can be achieved. The same design matrix was used to determine the best laser process parameters to be set. In this investigation, criteria were implemented to maximize the water contact angle, and the speed was also maximized so that surface texturing can be implemented on large surface area most efficiently within a practical period of time. Table 6 summarizes these criteria.

**Table 6:** Optimization criteria and their limits as used in this study

Parameter or response	Limits		Criterion	Importance
	*Lower*	*Upper*		
Laser power (W)	3	4	Is in range	3
Hatch distance (mm)	0.08	0.12	Is in range	3
Scan speed (mm/s)	3	5	Maximize	2
Arithmetic mean surface roughness S_a_ (µm)	0.509	0.658	Is in range	3
Maximum height surface roughness S_z_ (µm)	5.843	28.524	Is in range	3
Texture aspect ratio S_tr_ (µm)	0.028	0.058	Is in range	3
Water contact angle (deg)	97.95	143.78	Maximize	1

**Fig. 8:**
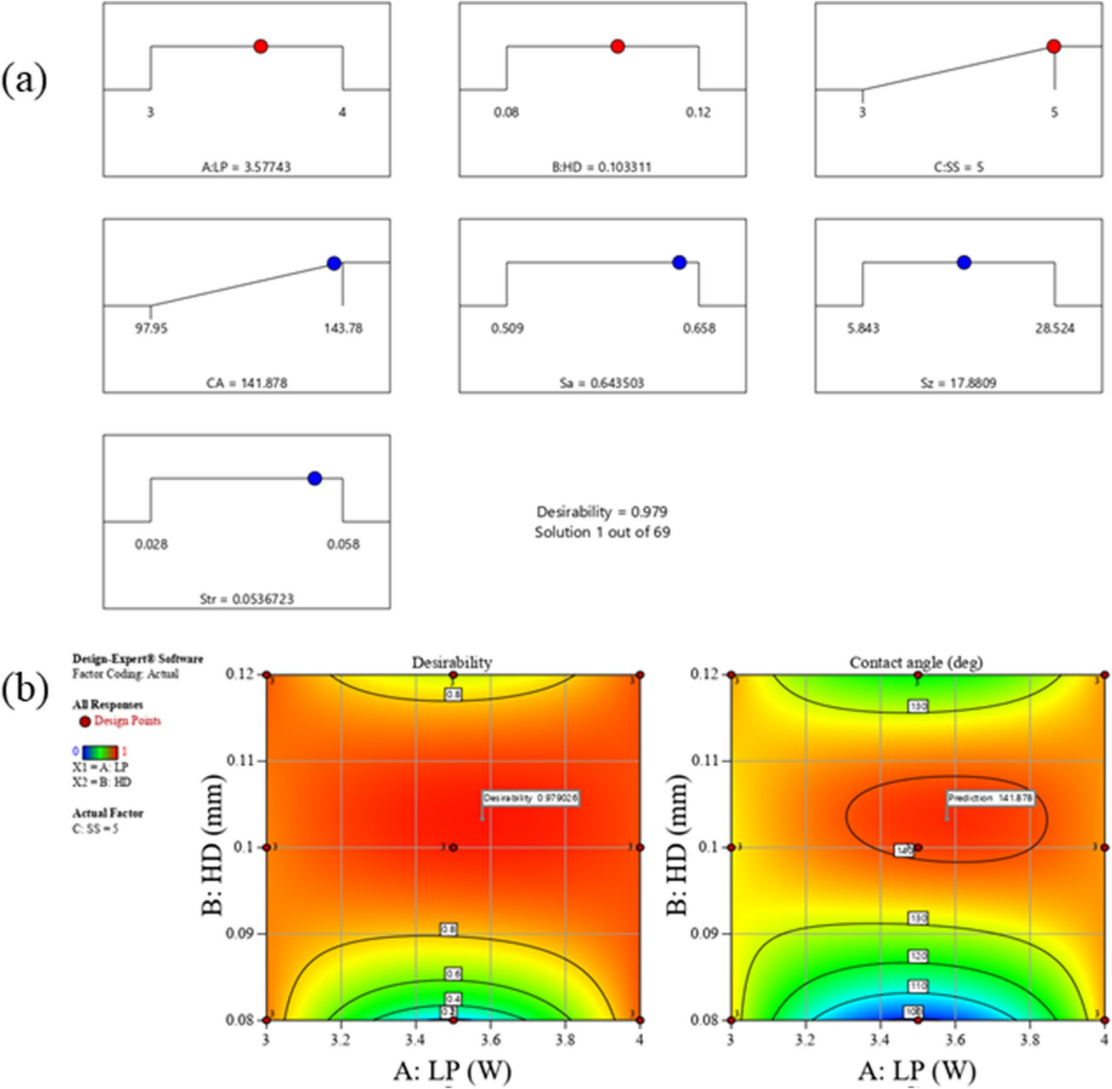
(**a**) Optimal laser process parameter conditions solution to produce a highly hydrophobic surface, with highest desirability of 98%, and (**b**) overlay contour plots solution for each response shows the region of optimal laser surface texturing condition based on the criterion to enhance hydrophobicity

There were 69 optimal solutions generated using the simple regression analysis method. Desirability values were calculated (see supplementary information) to find out the most optimum process parameters to produce hydrophobic surfaces. The solutions with the highest desirability are presented in Fig. 8.

#### Validation of the developed model

In order to validate this model, confirmation experiments were carried out on the new samples with the predicted parameters and the contact angle measurements were again characterized for contact angle as shown in the Table 7. The contact angle measurements are provided in the supplementary information. The validation results demonstrated that the models developed are quite accurate as the percentages of error in prediction were low.

**Table 7:** Validation test results for confirming the optimization model's predictive power

Solution no	Laser power (W)	Hatch distance (mm)	Scan speed (mm/s)		Contact angle
1(97.9% desirability)	3.577	0.103	5.000	Predicted	141.87
				Actual	141.25
				Error (%)	0.43
23(97% desirability)	3.406	0.104	5.000	Predicted	141.064
				Actual	137.34
				Error (%)	2.71
69(87.1% desirability)	3.000	0.092	5.000	Predicted	132.685
				Actual	124.15
				Error (%)	6.43

### Surface topology analysis of the laser treated area

The qualitative analysis was conducted on the samples using scanning electron microscope (SEM) of the laser microstructure aluminium alloy samples. Micro scale structure was successfully obtained on the 7075 alloy substrates, which was shown to play a major role on the different properties of the surfaces.

**Fig. 9:**
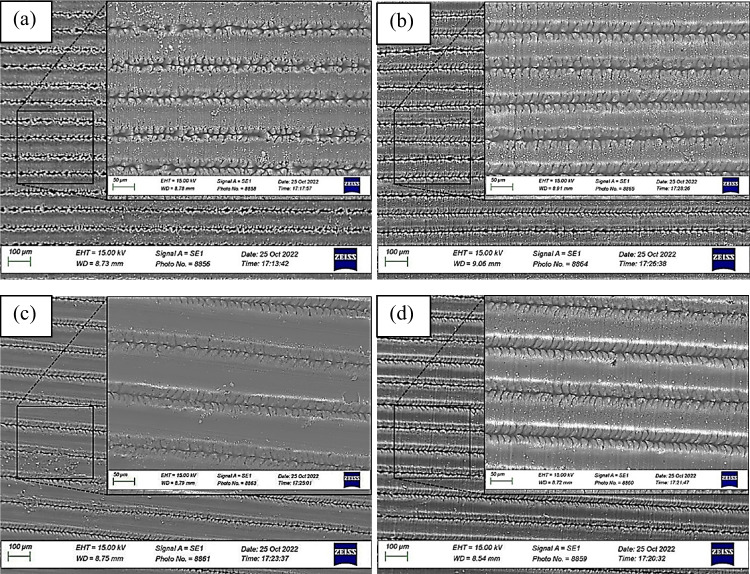
SEM images of the surface topology of the ultrafast laser structured 7075 for (**a**) sample 1, (**b**) sample 12, (**c**) sample 17, and (**d**) sample 23

Figure 9(a) illustrates the micro-trenches formed on sample 1 through laser surface texturing using a laser power of 3 W, a scan speed of 3 mm/s, and a hatch distance of 80 μm. The low power and slow scan speed resulted in the production of equally spaced, periodic trenches with a depth of 3.8 µm as measured by the profilometer data. Additionally, the laser surface texturing process resulted in a debris-covered machined area, as well as nano structures such as laser-induced periodic surface structures (LIPSS) on the layer, separated by uneven ridges and swells at the sub-micron level. These structures are primarily composed of oxides formed as a result of heating caused by laser irradiation. It is evident that the ridges and swells are periodically fabricated with substantial untreated areas in between the trenches.

In Fig. 9(b) shows sample 12 where the laser process parameters were set at 3.5 W laser power, 5 mm/s scan speed, and 80 μm hatch distance. It is observed that the trenches are closely packed and starting to merge, leaving very little untreated area between them, resulting in a highly rough surface. The average depth measured is quite high at 19 µm. By keeping the same fluence, a different structure with a higher hatch distance is seen in Fig. 9(c) (sample 17). The laser process parameters were set at 3.5 W laser power, 4 mm/s scan speed, and 0.12 mm. It is observed that the trenches are parallel to each other with untreated surface areas. The ridges and swells are periodic but less than those in Fig. 9(a). The effect of low scan speed is observed when comparing Figs. 9(b) and (c).

Figure 9(d) shows sample 23 where it can be observed that the trenches are equally spaced with a hatch distance of 100 μm and were produced using laser processing parameters of 4 W and a scanning speed of 4 mm/s. The non-processed area observed is smaller than that in Fig. 9(c). It is also observed that there are material deposits on the peaks, which form periodic surface structures that could be the result of oxidation caused during laser processing and aging. These periodic nano and micro surface structures act as porous surfaces that could trap air between the gaps, leading to highly hydrophobic surfaces. The maximum depth measured from the peak is 21 µm.

#### Surface chemistry analysis

The change in the wetting properties of the laser processed aluminium alloy samples can also be attributed to the changes in the surface chemistry of the samples. This can occur due to exposure of the samples to the atmospheric conditions, leading to oxidation of the aluminium alloy and formation of Al_2_O_3_ on the surface [37]. Additionally, a carbonaceous layer can also form on the textured surface, leading to the formation of non-polar regions and an increase in hydrophobicity. Aluminium alloy 7075 is a zinc-based alloy and when exposed to atmospheric aging, it leads to the formation of ZnO, which are also non-polar regions and may lead to an increase in hydrophobicity. To verify these theories [37, 39], X-ray photoelectron spectroscopy (XPS) measurements were performed on the surface of the laser-treated samples to determine their chemical composition. The XPS measurements were performed on the same four samples on the first day and after 60 days, when the samples turned to a hydrophobic state. This is performed to understand the change in chemical composition of the surface, and how it affects the wettability of the surface over the time.

Table 8 shows the results at day 1 and day 60 of the XPS analysis conducted in atomic percentage for the samples 1, 12, 17, and 23, as well as the recorded water contact angles for these samples, at day 1 and day 60.

**Table 8:** Atomic composition of laser surface structured samples determined by XPS on the day of and 60 days after laser treatment

Sample No	WCA day 1 (deg)	WCA day 60 (deg)	Elements	Day 1 atomic %	Day 60 atomic %
1	67.19	141.5	Al	23.99	28.48
			C	34.14	9.04
			Zn	0.97	4.24
			O	40.90	58.24
12	18.14	97	Al	5.65	29.02
			C	82.41	11.82
			Zn	0.12	3.23
			O	11.81	55.93
17	11.35	123.5	Al	16.78	24.91
			C	41.92	15.86
			Zn	0.56	4.24
			O	40.75	54.99
23	21.31	141.72	Al	21.73	28.37
			C	41.90	11.76
			Zn	0.77	4.26
			O	35.60	55.64

In this study, it was observed that laser treatment led to a decrease in carbon content from 34 to 9% in sample 1, 82 to 11% in sample 12, from 41 to 15% in sample 10, and 42 to 12% in sample 23, compared to unchanged in the unprocessed metal with carbon content of 87%. Moreover, an increase in oxygen and polar components results in the formation of oxides such as Al_2_O_3_ and ZnO.

**Fig. 10:**
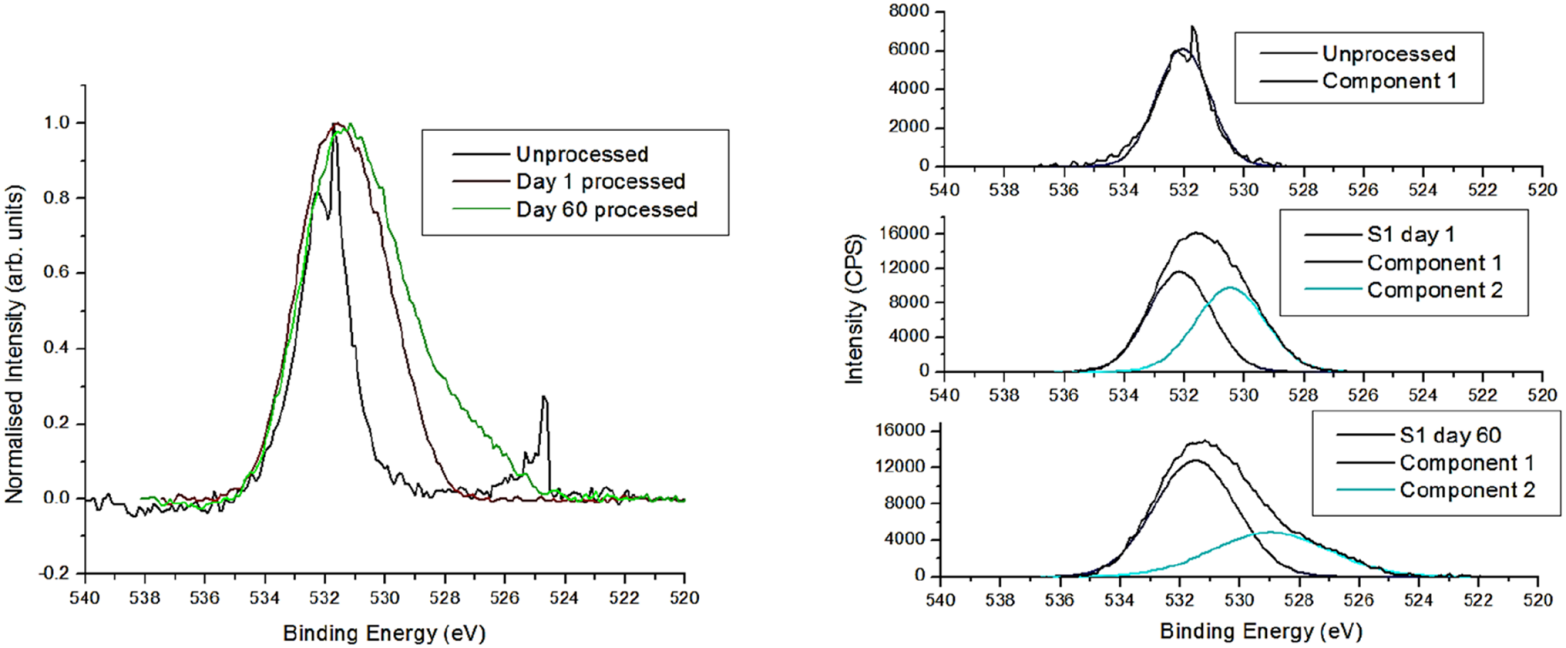
XPS spectra of the O 1 s peak for unprocessed metal, and laser processed samples on the same day and the sixtieth day after laser processing for sample 1 (left), and with component fits for each plot (right)

A comparison of the O 1 s peak for the unprocessed metal and a typical day 1 and day 60 processed sample (sample 1) is shown in Fig. 10. While some oxygen in the results may be due to surface adsorbates, the extension into lower binding energy in the O 1 s peak is consistent with the presence of metal oxides, with the presence of metal oxide increasing strongly after processing and extending lower in aged samples. Deconvolved components were fit using Gaussian–Lorentzian functions, with additional components only included when they reduced the RMS of the fit. For the unprocessed sample, a single component at 532.0 eV gave the best fit, with any lower binding energy component below the limit of detection, indicating minimal metal oxides. A first component at ~ 532 eV with a second, lower binding energy component (529.6–530.8 eV) created a better fit for all processed samples. These results support strongly that the laser process induces metal oxides at the surface relative to the unprocessed sample. In the unprocessed metal, the Zn 2p peak was below the limit of detection, while 0.6–4.3% Zn was found for the processed samples. Due to the close binding energies of Zn and ZnO 2p peaks (1021.7 and ~ 1022 eV), these were narrow peaks which could not be deconvolved into metallic and oxide components. For the Al 2p peak, the binding energy for metallic Al (72.6 eV) and Al oxides (74.6—75.6 eV) are more distinct. The Al peaks for all samples were centred at higher binding energies consistent with Al oxide. Fitting components, best fits were found for two components in all cases, with the smaller metallic low binding energy component (71.1–73.8 eV) having relative area for day 1 and day 60 of 4 and 30% for sample 1, 28 and 43% for sample 12, 38 and 42% for sample 17, and 38 and 36% for sample 23, compared to the higher binding energy component. For sample 23 the oxide level stays similar, while the result for the other samples shows a more metal rich signal for the aged samples, suggesting the Al oxide may diminish over time. As the oxygen signal does not decrease for the aged samples, this could suggest an increase in ZnO for the aged samples; however, this cannot be confirmed due to the close positions of the Zn and ZnO 2p peaks. ZnO thin films have established hydrophobic properties, and increasing surface levels of ZnO could cause the increased hydrophobicity in the aged samples [50–52].

The formation of zinc oxide and aluminium oxide is found to be largely dependent on laser process parameters, which significantly impacted the wettability of the surface. A lower percentage of zinc is found to correspond with a lower water contact angle. For instance, sample 12 (fabricated using laser process parameters 3.5 W laser power, 0.08 mm hatch distance, and 5 mm/s scan speed) exhibited the lowest contact angle of 97° and the lowest percentage of zinc at 3.23%. Conversely, the highest water contact angle was observed in sample 23 (fabricated using laser process parameters 4 W laser power, 0.1 mm hatch distance and 4 mm/s scan speed) and the highest percentage of zinc. Additionally, the percentage of Al_2_O_3_ on the surface was found to affect wettability, as observed by the difference in contact angles between sample 17 (with contact angle of 123.5° and manufactured using laser process parameters 3.5 W laser power, 0.12 mm hatch distance, and 4 mm/s scan speed) and sample 1 (with contact angle of 141.5° and manufactured using laser process parameters 3 W laser power, 0.08 mm hatch distance, and 3 mm/s scan speed) despite similar zinc percentages, which is in line with previous findings [21].

## Discussion

The results of this study indicate that laser texturing using ultrafast femtosecond laser can be an effective method for modifying the surface properties of aluminium alloy 7075 materials despite various material challenges such as high reflectivity and high thermal conductivity [53]. The changes observed in surface morphology, surface roughness, wettability, and elemental composition in this study suggest that femtosecond laser texturing treatment can be used to tailor the surface properties of materials for specific applications.

Moreover, the optimization of the process parameters of the laser plays a key role in finding out the best process parameter to get larger hydrophobicity, as greater hydrophobicity enhances the corrosion resistance and fouling resistance. The findings of this study also provide insights into the effects of femtosecond laser treatment on the surface properties of the material and fill the research gap in the research paper by Milles et al. [21] on understanding the time-dependent evolution of wetting properties and finding out the relationship of the laser process parameters and wetting properties. Moreover, this paper also provides an understanding of the surface chemical changes observed especially focusing on the enrichment of formation of zinc oxide on the surface which is one of the secondary reasons for hydrophobicity. Understanding surface chemical changes can help researchers optimize the laser texturing process to achieve desired surface properties.

The findings of this study have significant implications for various industries that require tailored surface properties for improved material performance in specific applications. For example, the marine industry can benefit from femtosecond laser texturing to enhance the corrosion resistance of aluminium alloys used in ship components. Similarly, the medical industry can benefit from tailored surface properties to improve the biocompatibility of implantable medical devices.

## Conclusion

In conclusion, this study successfully demonstrated the use of ultrafast pulse laser surface machining to produce hydrophobic surfaces on aluminium alloy (7075) by optimizing the laser process parameters. Through a full factorial design using the response surface method, the effects of changes in roughness attributes and wettability were studied and statistically significant mathematical models were developed to describe the relationships between laser process parameters and functional properties. The most influential process parameters for controlling surface roughness and wettability were identified and a combination of laser power, hatch distance, and scanning speed produced extremely hydrophobic surfaces with a maximum achieved water contact angle of 142°. Additionally, we conducted a qualitative analysis of the micron-level surface structures using scanning electron microscopy, finding highly defined surface structures with recast layers adjacent to the laser textured area. The surface chemical analysis on this surface revealed the formation of a substitution of carbides with oxides and ZnO which leads to increased hydrophobicity due to changes in surface chemistry. In conclusion, this study highlights the method to optimize the main laser input parameters for producing highly hydrophobic surfaces on aluminium 7075 alloy. In future work, different scan strategies can be implemented for specific applications to produce corrosion-resistant, anti-fouling, and anti-icing surfaces.

### Supplementary Information

Below is the link to the electronic supplementary material.

Supplementary file1 (DOCX 3609 KB)
