# Supplementary material for: Parametric investigation of ultrashort pulsed laser surface texturing on aluminium alloy 7075 for hydrophobicity enhancement
Source: Int J Adv Manuf Technol. 2024 Jan 13;130(9-10):4169–86. doi: 10.1007/s00170-024-12971-8 (PMC10810958; doi:10.1007/s00170-024-12971-8)
Supplement: Supplementary file 1 — Supplementary file1 (DOCX 3609 KB) [file 170_2024_12971_MOESM1_ESM.docx]

**Parametric Investigation of Ultrashort Pulsed Laser Surface Texturing on Aluminum Alloy 7075 for Hydrophobicity Enhancement**

Abhijit Cholkar ^a,b,d^*, Suman Chatterjee ^a,b,d^, Feljin Jose ^c^, Robert O'Connor ^b,c^, Éanna McCarthy ^a,b,c^ , Nick Weston ^e^ , David Kinahan ^a,b,d^ , Dermot Brabazon ^a,b,d^

^a^ I-Form, Advanced Manufacturing Research Centre, Dublin City University, Glasnevin, Dublin, Ireland

^b^ Advanced Processing Technology Research Centre, School of Mechanical and Manufacturing Engineering, Dublin City University, Glasnevin, Dublin , Ireland

^c^ School of Physical Sciences, Dublin City University, Glasnevin, Dublin , Ireland

^d^ DCU Water Institute, Dublin City University, Glasnevin, Dublin , Ireland

^e^ Renishaw Edinburgh, Riccarton, Edinburgh, EH14 4AP, UK

*Corresponding Author: Abhijit Cholkar

School of Mechanical and Manufacturing Engineering,

Dublin City University, Ireland

Email: [abhijit.cholkar2@mail.dcu.ie](mailto:abhijit.cholkar2@mail.dcu.ie)

# Supplementary Information

# Beam Profile measured by beam profiler.


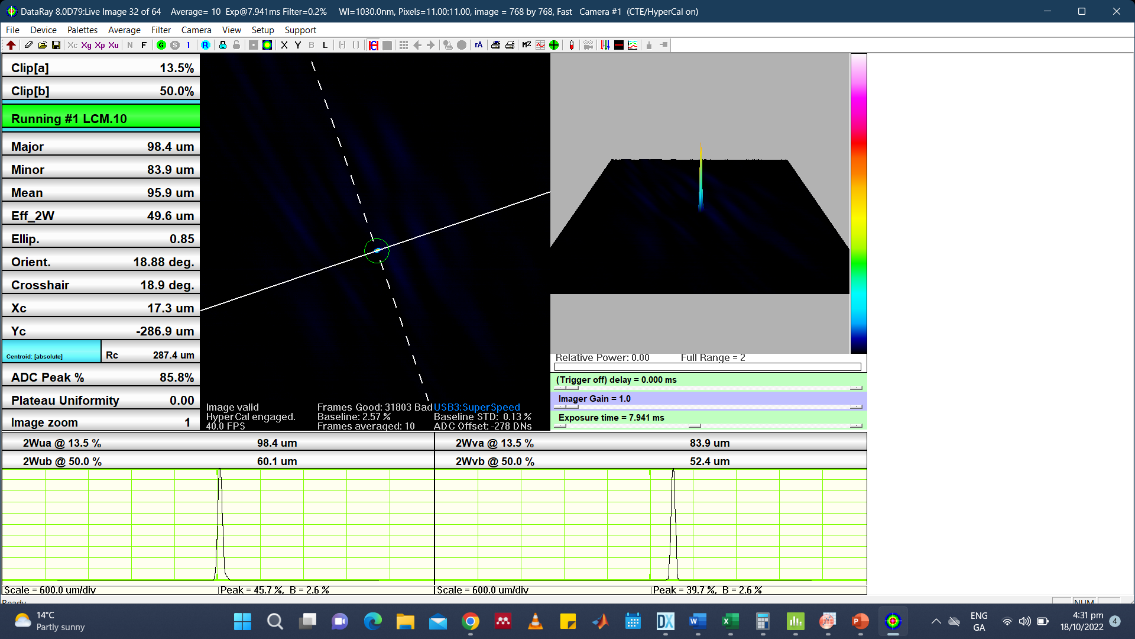


**Figure 1** Beam profile at focus position

| Samples | LP | HD | SS | CA | Sa | Sz | Str |
| --- | --- | --- | --- | --- | --- | --- | --- |
| 1 | 3 | 0.08 | 3 | 141.51 | 0.93 | 21.6 | 0.439 |
| 2 | 3 | 0.08 | 4 | 140.46 | 0.91 | 18.4 | 0.452 |
| 3 | 3 | 0.08 | 5 | 137.77 | 0.89 | 14.3 | 0.43 |
| 4 | 3 | 0.1 | 3 | 138.45 | 0.78 | 18.6 | 0.049 |
| 5 | 3 | 0.1 | 4 | 136.7 | 0.77 | 13.3 | 0.037 |
| 6 | 3 | 0.1 | 5 | 137.34 | 0.74 | 7.4 | 0.03 |
| 7 | 3 | 0.12 | 3 | 142.19 | 1.81 | 21.4 | 0.352 |
| 8 | 3 | 0.12 | 4 | 135.13 | 1.38 | 14 | 0.032 |
| 9 | 3 | 0.12 | 5 | 136.28 | 0.9 | 9.3 | 0.042 |
| 10 | 3.5 | 0.08 | 3 | 109.06 | 2.82 | 21.5 | 0.45 |
| 11 | 3.5 | 0.08 | 4 | 138.73 | 1.9 | 19.9 | 0.448 |
| 12 | 3.5 | 0.08 | 5 | 97.95 | 1.28 | 17.3 | 0.448 |
| 13 | 3.5 | 0.1 | 3 | 137 | 1.57 | 21.8 | 0.152 |
| 14 | 3.5 | 0.1 | 4 | 135.59 | 1.43 | 13.1 | 0.437 |
| 15 | 3.5 | 0.1 | 5 | 135.7 | 1.04 | 9.6 | 0.511 |
| 16 | 3.5 | 0.12 | 3 | 133.59 | 1.94 | 25.6 | 0.066 |
| 17 | 3.5 | 0.12 | 4 | 123.54 | 1.36 | 22.3 | 0.509 |
| 18 | 3.5 | 0.12 | 5 | 114.01 | 0.97 | 13.6 | 0.535 |
| 19 | 4 | 0.08 | 3 | 133.33 | 2.49 | 26.9 | 0.085 |
| 20 | 4 | 0.08 | 4 | 122.07 | 2.34 | 21.3 | 0.134 |
| 21 | 4 | 0.08 | 5 | 135.93 | 2.03 | 17.5 | 0.504 |
| 22 | 4 | 0.1 | 3 | 141.72 | 2.74 | 29.2 | 0.454 |
| 23 | 4 | 0.1 | 4 | 138.75 | 1.26 | 21.1 | 0.289 |
| 24 | 4 | 0.1 | 5 | 137.31 | 0.95 | 18.8 | 0.51 |
| 25 | 4 | 0.12 | 3 | 137.01 | 3 | 23.5 | 0.049 |
| 26 | 4 | 0.12 | 4 | 138.55 | 2.98 | 22.1 | 0.439 |
| 27 | 4 | 0.12 | 5 | 138.39 | 2.33 | 21 | 0.522 |
| 28 | 3 | 0.08 | 3 | 141.1 | 0.89 | 18.3 | 0.439 |
| 29 | 3 | 0.08 | 4 | 139.21 | 0.88 | 16.4 | 0.424 |
| 30 | 3 | 0.08 | 5 | 132.17 | 0.77 | 13.8 | 0.046 |
| 31 | 3 | 0.1 | 3 | 136.73 | 0.7 | 21.5 | 0.035 |
| 32 | 3 | 0.1 | 4 | 137.07 | 0.71 | 17.5 | 0.066 |
| 33 | 3 | 0.1 | 5 | 134.79 | 0.68 | 7.6 | 0.03 |
| 34 | 3 | 0.12 | 3 | 135.07 | 1.73 | 22 | 0.048 |
| 35 | 3 | 0.12 | 4 | 137.12 | 1.37 | 16.3 | 0.031 |
| 36 | 3 | 0.12 | 5 | 136.69 | 0.84 | 11.2 | 0.053 |
| 37 | 3.5 | 0.08 | 3 | 103.49 | 1.91 | 21.7 | 0.452 |
| 38 | 3.5 | 0.08 | 4 | 132.44 | 2.63 | 19 | 0.45 |
| 39 | 3.5 | 0.08 | 5 | 99.15 | 1.28 | 17.5 | 0.455 |
| 40 | 3.5 | 0.1 | 3 | 136.56 | 1.43 | 19.7 | 0.088 |
| 41 | 3.5 | 0.1 | 4 | 129.6 | 1.14 | 13 | 0.433 |
| 42 | 3.5 | 0.1 | 5 | 143.08 | 1.09 | 9.1 | 0.494 |
| 43 | 3.5 | 0.12 | 3 | 124.58 | 1.01 | 21.8 | 0.059 |
| 44 | 3.5 | 0.12 | 4 | 123.16 | 1.98 | 18.4 | 0.423 |
| 45 | 3.5 | 0.12 | 5 | 122.7 | 1.86 | 16.3 | 0.512 |
| 46 | 4 | 0.08 | 3 | 127.93 | 2.44 | 24.5 | 0.092 |
| 47 | 4 | 0.08 | 4 | 122.2 | 2.33 | 15.6 | 0.107 |
| 48 | 4 | 0.08 | 5 | 136.48 | 1.18 | 21.3 | 0.501 |
| 49 | 4 | 0.1 | 3 | 137.07 | 2.13 | 28.8 | 0.449 |
| 50 | 4 | 0.1 | 4 | 133.58 | 1.24 | 17.7 | 0.438 |
| 51 | 4 | 0.1 | 5 | 136.74 | 0.9 | 12.2 | 0.527 |
| 52 | 4 | 0.12 | 3 | 134.33 | 1.95 | 22.8 | 0.067 |
| 53 | 4 | 0.12 | 4 | 130.99 | 2.39 | 22.4 | 0.084 |
| 54 | 4 | 0.12 | 5 | 138.4 | 2.47 | 21.1 | 0.52 |
| 55 | 3 | 0.08 | 3 | 141.73 | 0.8 | 18.4 | 0.446 |
| 56 | 3 | 0.08 | 4 | 138.27 | 0.87 | 16.6 | 0.428 |
| 57 | 3 | 0.08 | 5 | 127.23 | 0.89 | 13.6 | 0.248 |
| 58 | 3 | 0.1 | 3 | 130.99 | 0.67 | 10.2 | 0.027 |
| 59 | 3 | 0.1 | 4 | 138.8 | 0.69 | 20.3 | 0.037 |
| 60 | 3 | 0.1 | 5 | 130.57 | 0.69 | 9.7 | 0.034 |
| 61 | 3 | 0.12 | 3 | 131.09 | 4.06 | 15.9 | 0.045 |
| 62 | 3 | 0.12 | 4 | 133.19 | 2.41 | 11.1 | 0.029 |
| 63 | 3 | 0.12 | 5 | 137.89 | 1.38 | 24.5 | 0.053 |
| 64 | 3.5 | 0.08 | 3 | 112.2 | 0.8 | 19.4 | 0.439 |
| 65 | 3.5 | 0.08 | 4 | 129.45 | 2.91 | 22 | 0.449 |
| 66 | 3.5 | 0.08 | 5 | 103.25 | 2.16 | 17.1 | 0.437 |
| 67 | 3.5 | 0.1 | 3 | 135.01 | 1.42 | 9.3 | 0.084 |
| 68 | 3.5 | 0.1 | 4 | 126.41 | 1.12 | 13.8 | 0.437 |
| 69 | 3.5 | 0.1 | 5 | 143.78 | 1.16 | 19.8 | 0.501 |
| 70 | 3.5 | 0.12 | 3 | 133.96 | 1.87 | 13.6 | 0.059 |
| 71 | 3.5 | 0.12 | 4 | 122.71 | 1.18 | 28.5 | 0.422 |
| 72 | 3.5 | 0.12 | 5 | 121.22 | 0.97 | 17.7 | 0.545 |
| 73 | 4 | 0.08 | 3 | 126.97 | 2.53 | 18.5 | 0.49 |
| 74 | 4 | 0.08 | 4 | 119.62 | 2.2 | 23.3 | 0.114 |
| 75 | 4 | 0.08 | 5 | 136.11 | 2.1 | 18 | 0.51 |
| 76 | 4 | 0.1 | 3 | 136.56 | 2.1 | 17.7 | 0.466 |
| 77 | 4 | 0.1 | 4 | 134.89 | 1.26 | 22.4 | 0.437 |
| 78 | 4 | 0.1 | 5 | 133.61 | 0.89 | 18.6 | 0.527 |
| 79 | 4 | 0.12 | 3 | 133.42 | 2.45 | 22.5 | 0.051 |
| 80 | 4 | 0.12 | 4 | 134.44 | 2.22 | 22.4 | 0.116 |
| 81 | 4 | 0.12 | 5 | 137.69 | 1.69 | 21 | 0.51 |

**Table 1**: Pearson correlation data


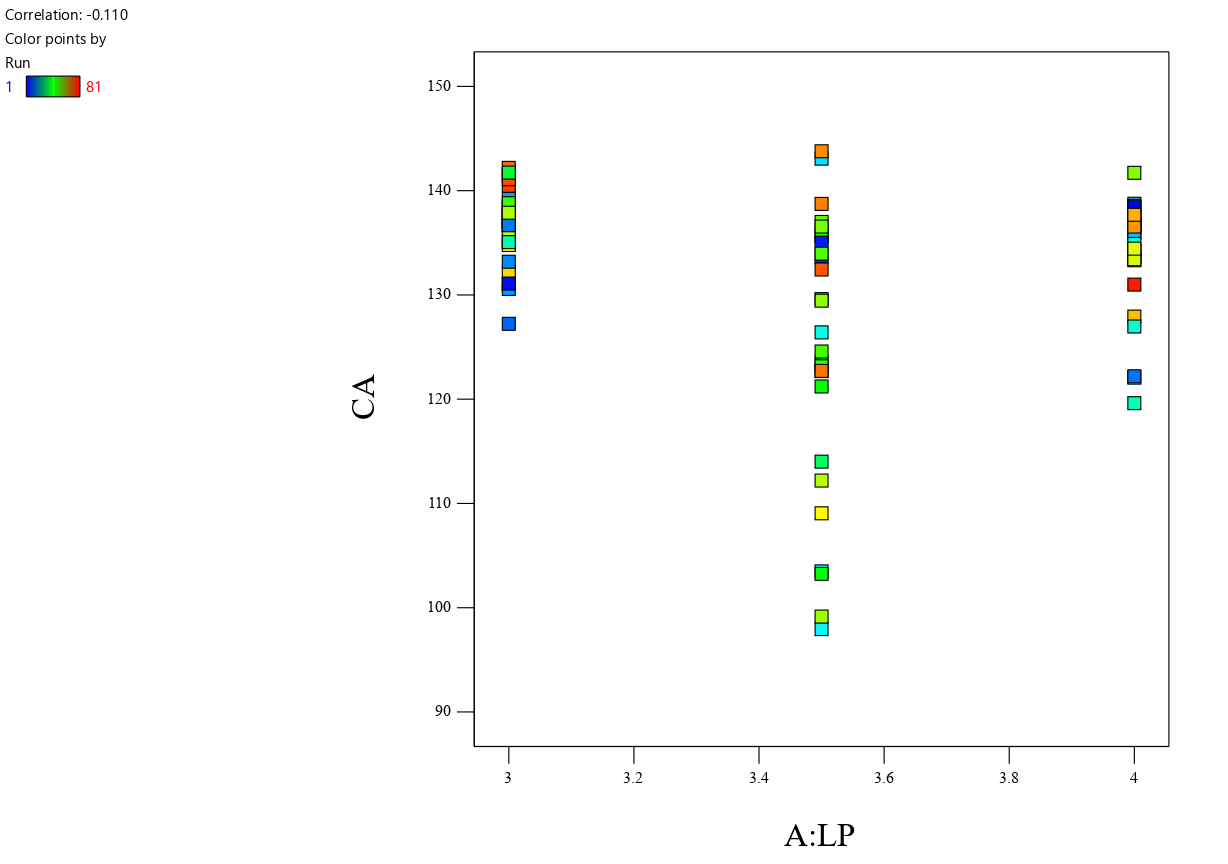

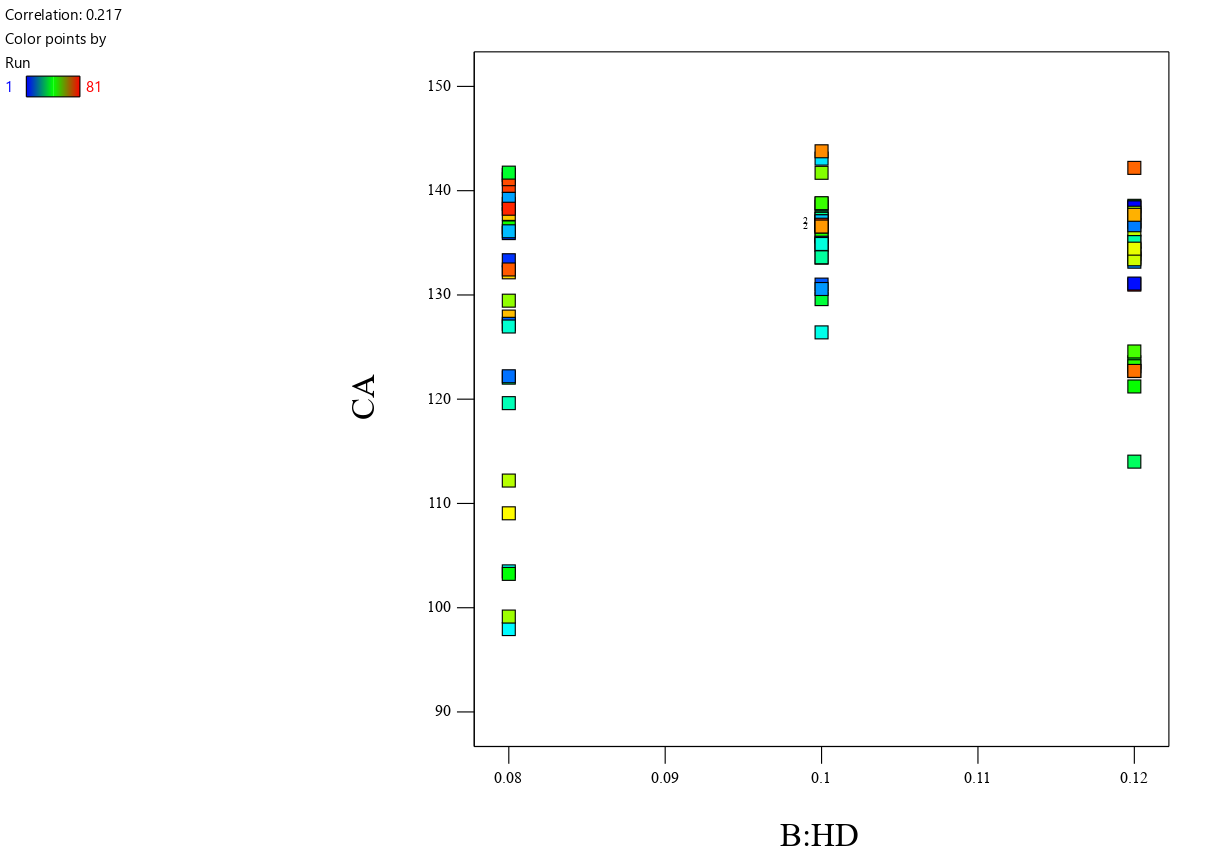


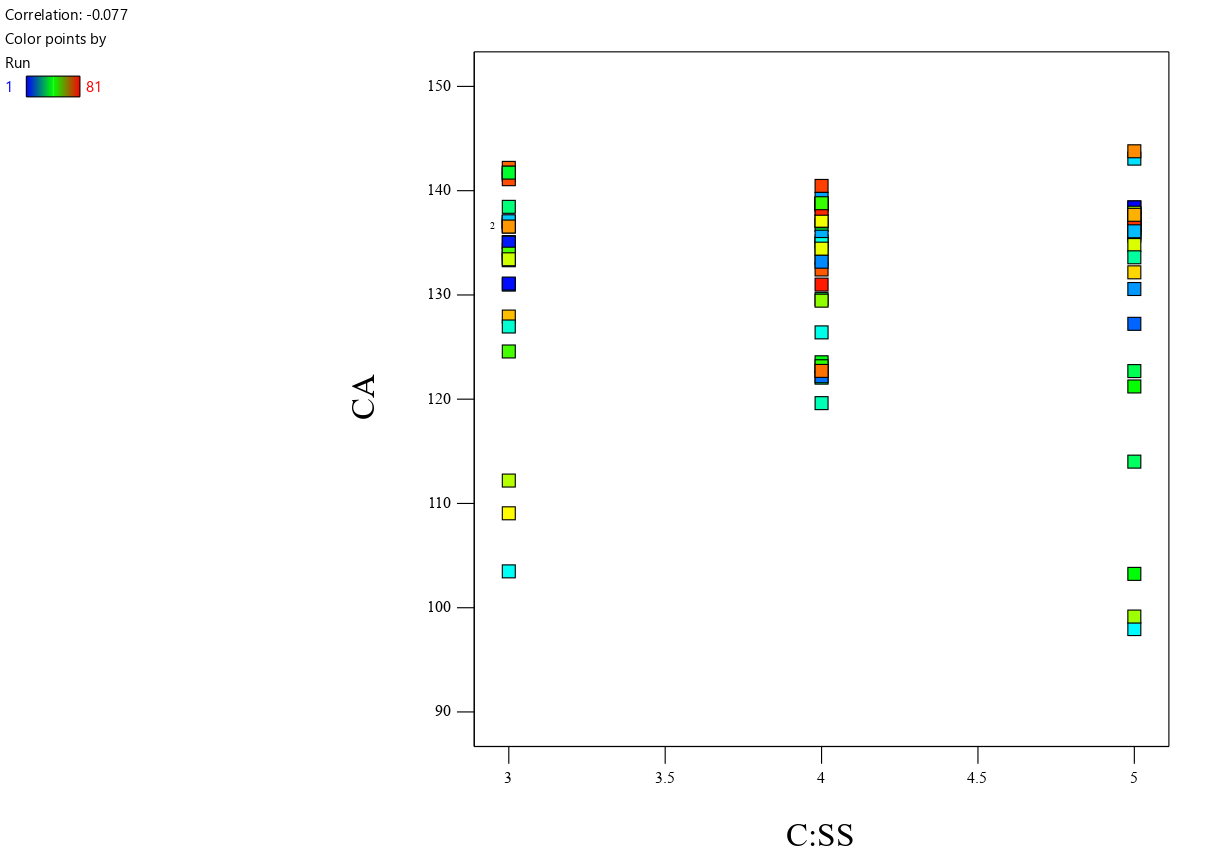


**Figure 2:** Scatter plots showing the correlation of contact angle with laser process parameters such as laser power, hatch distance and scan speed.


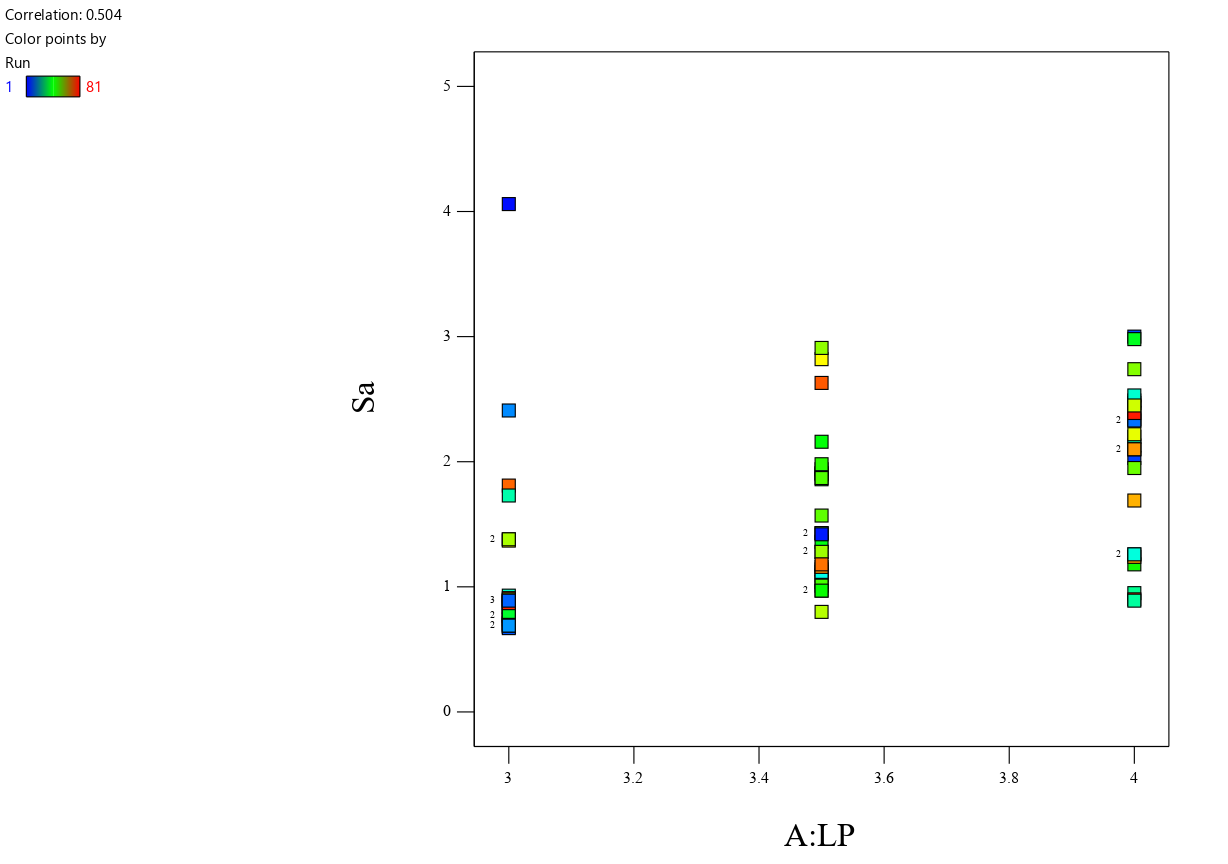

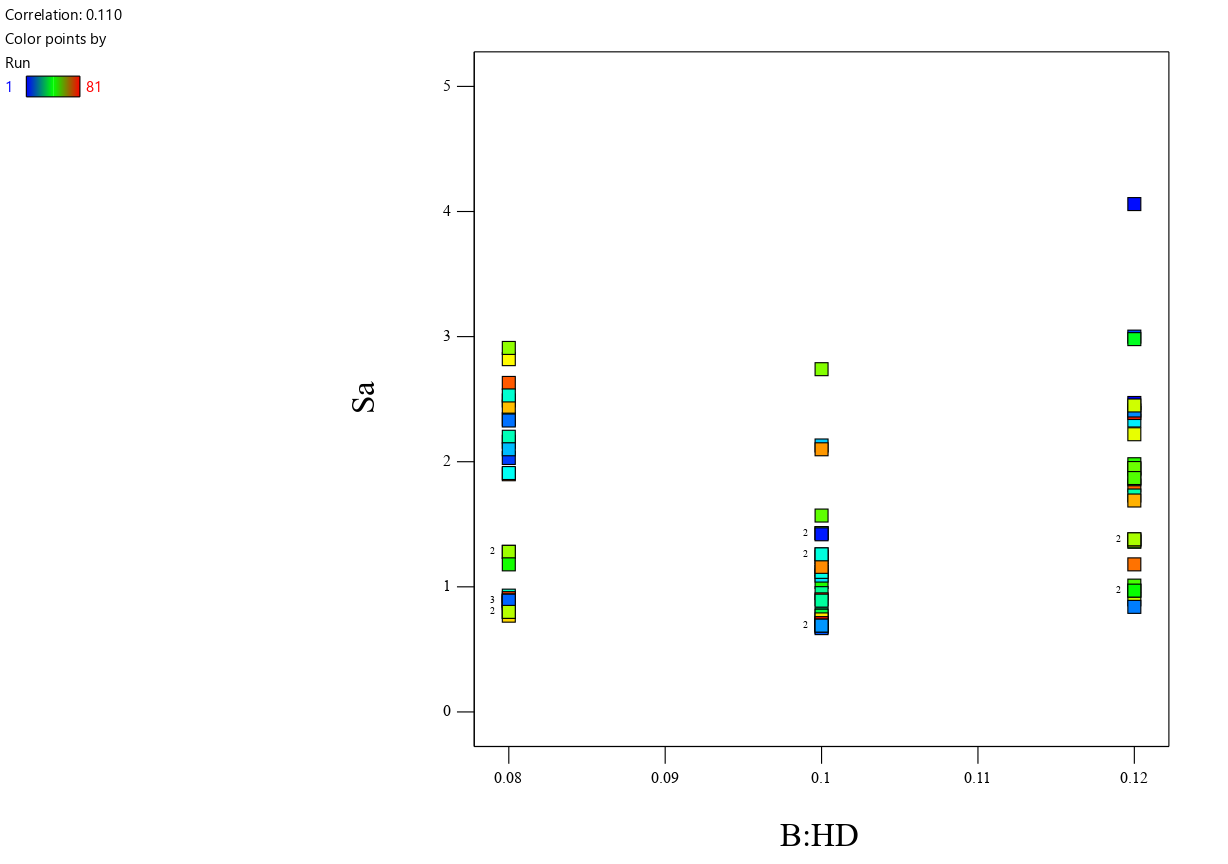


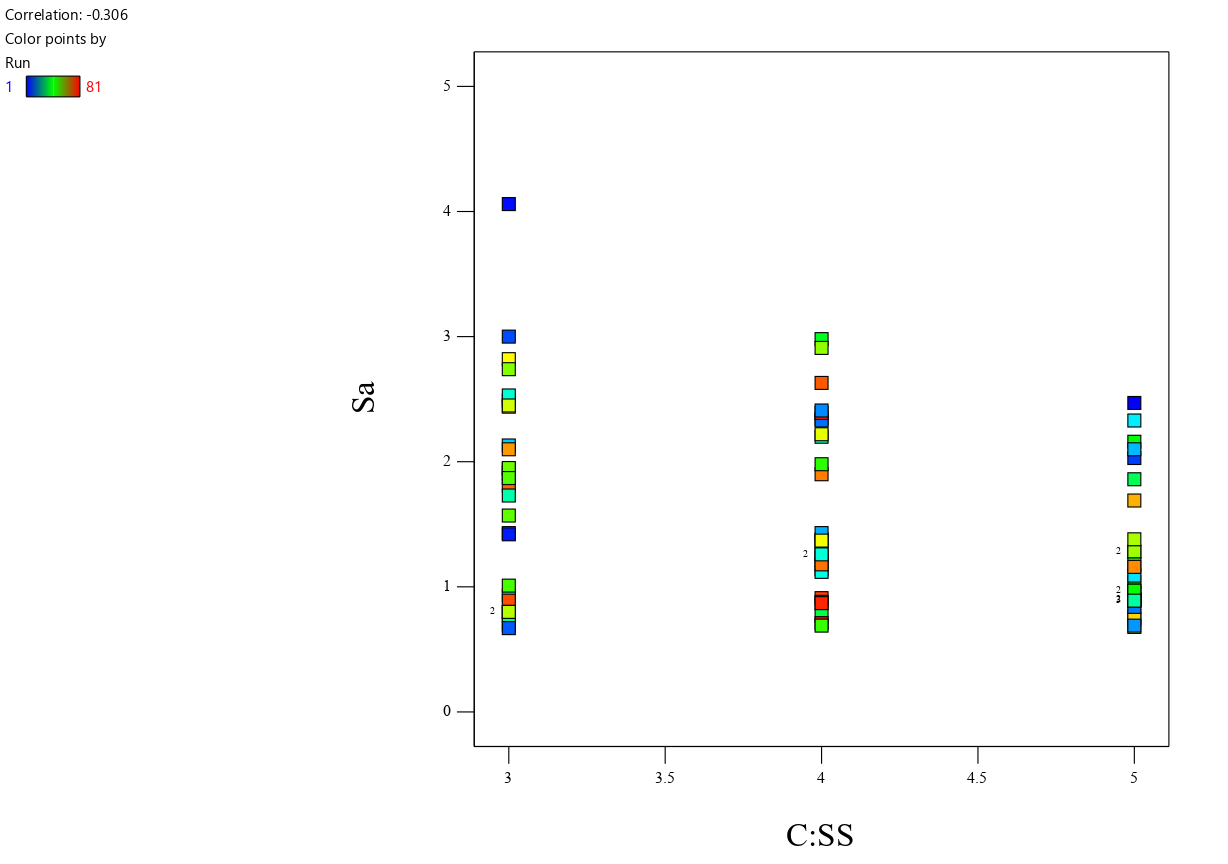


**Figure 3:** Scatter plots showing the correlation of average surface roughness (S_a_) with laser process parameters such as laser power, hatch distance and scan speed.


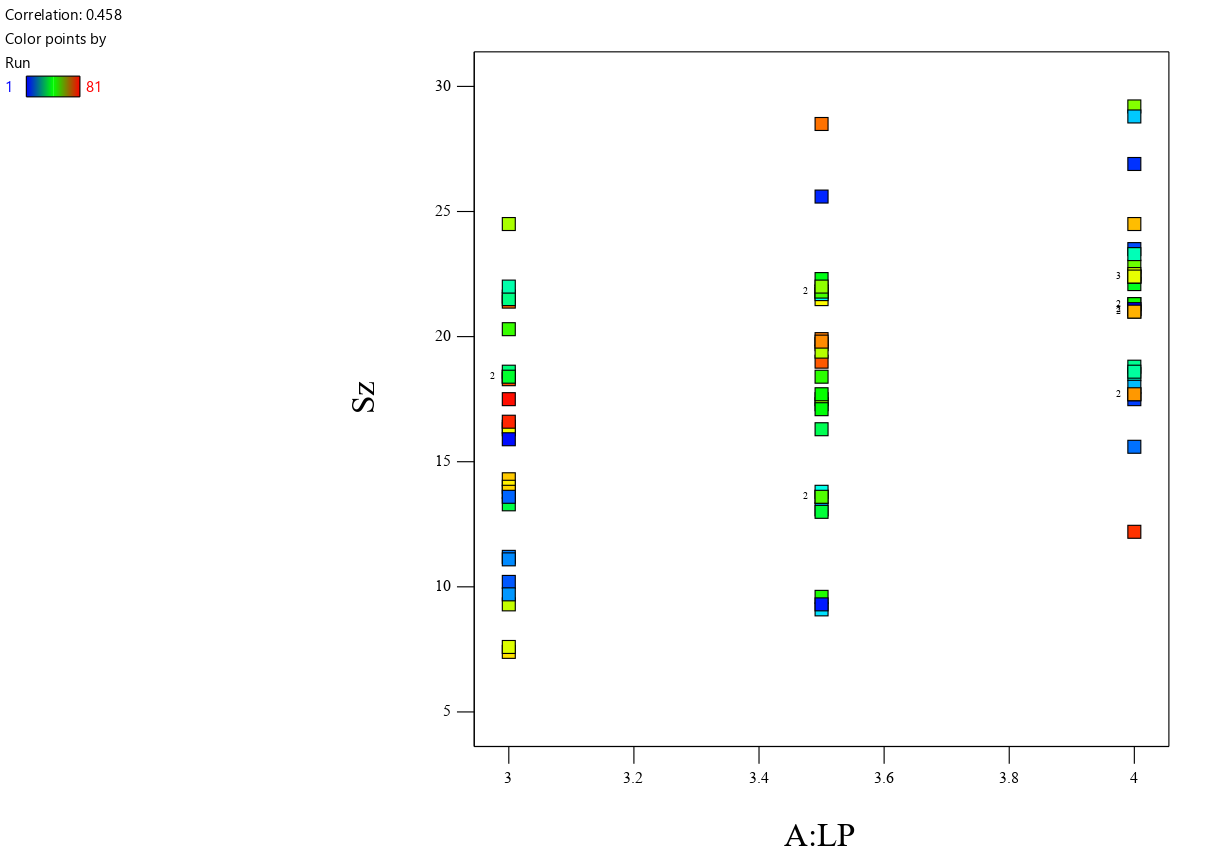

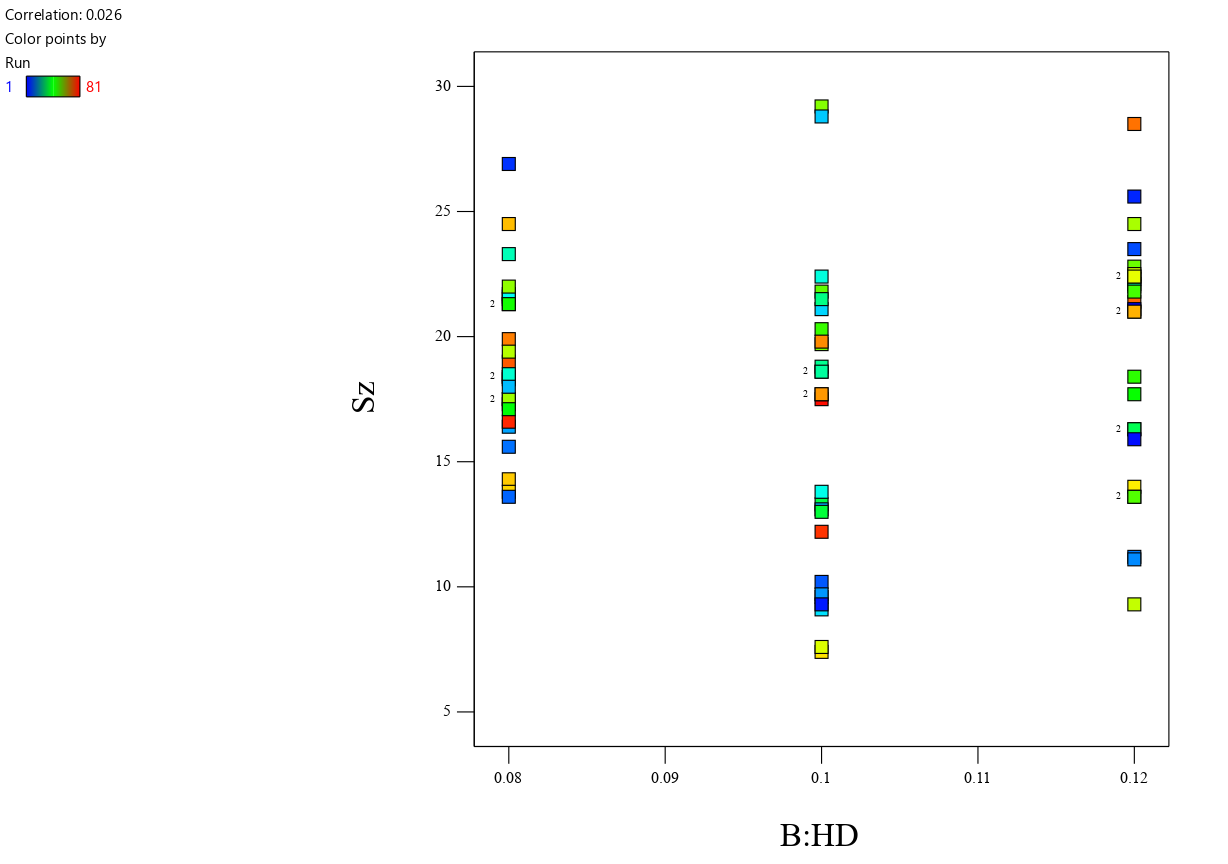


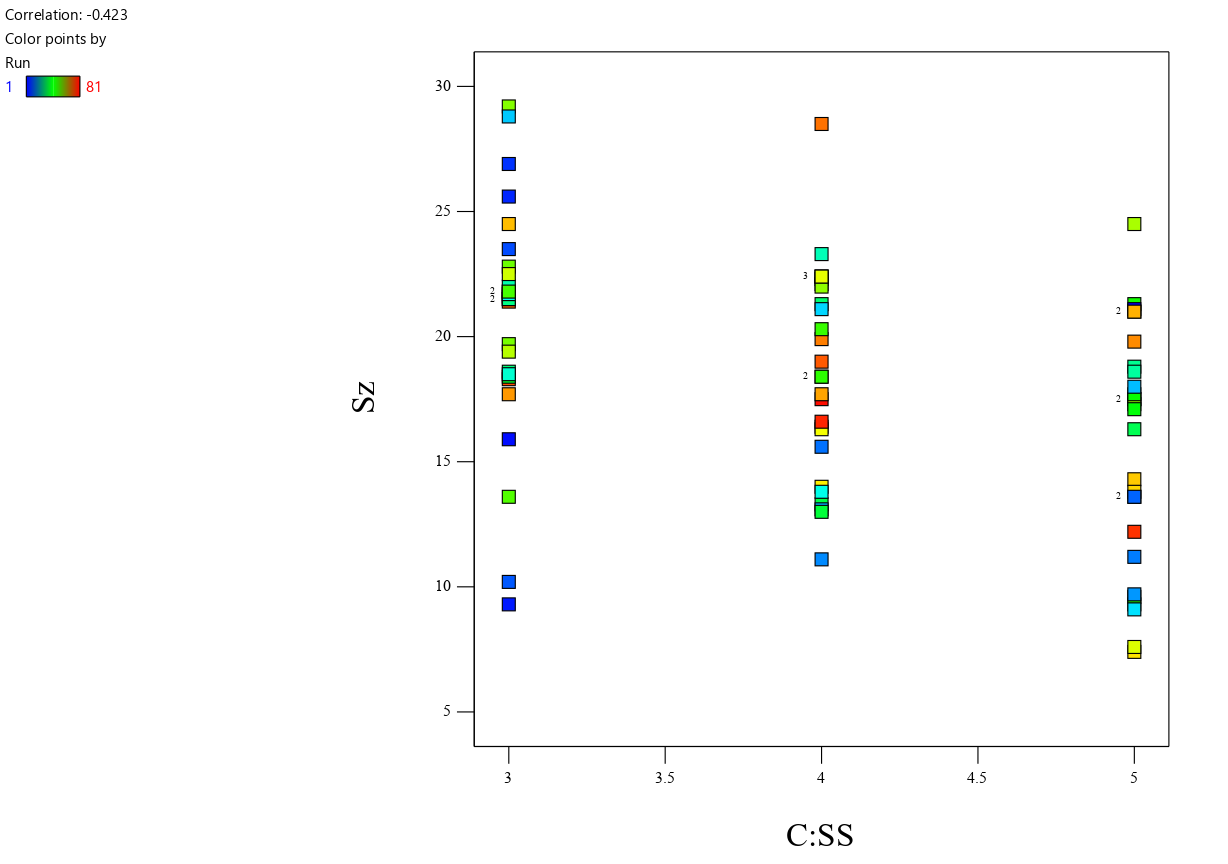


**Figure 4:** Scatter plots showing the correlation of maximum depth surface roughness (S_z_) with laser process parameters such as laser power, hatch distance and scan speed.


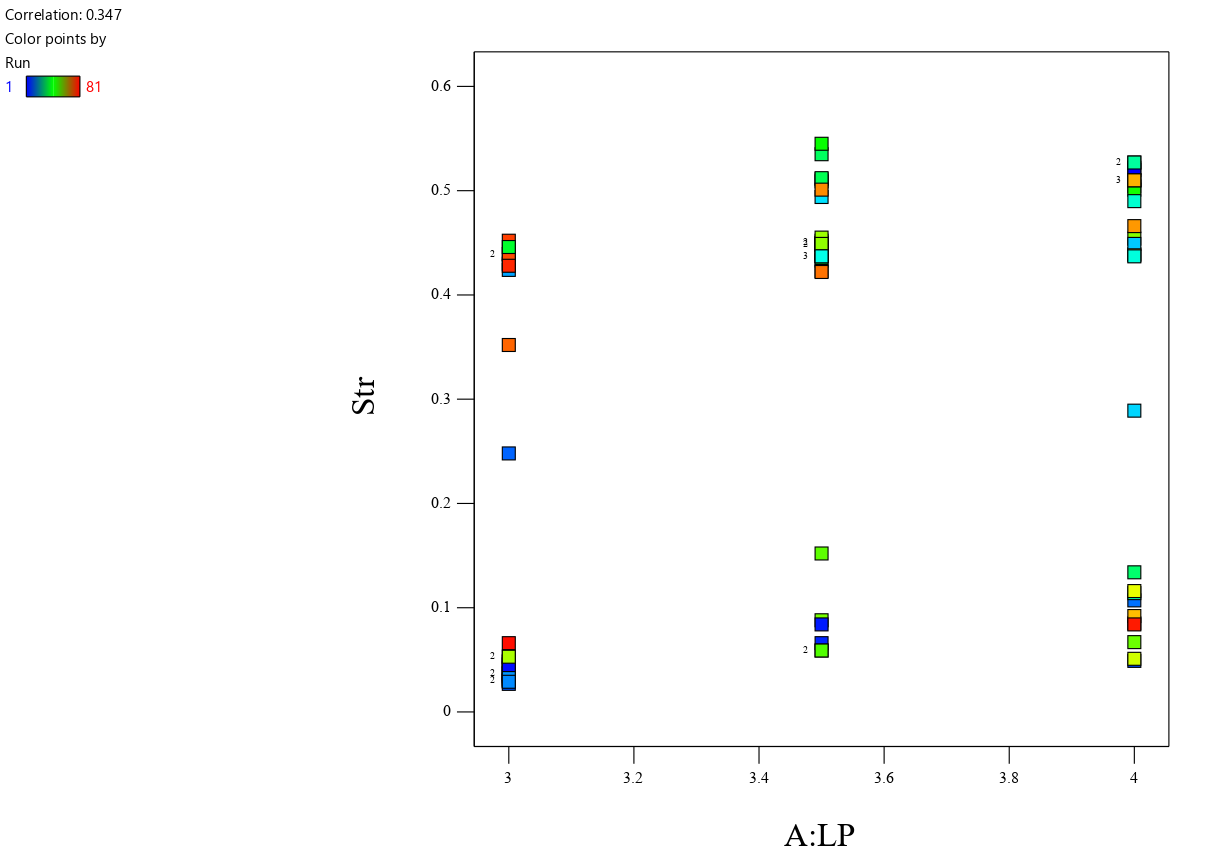

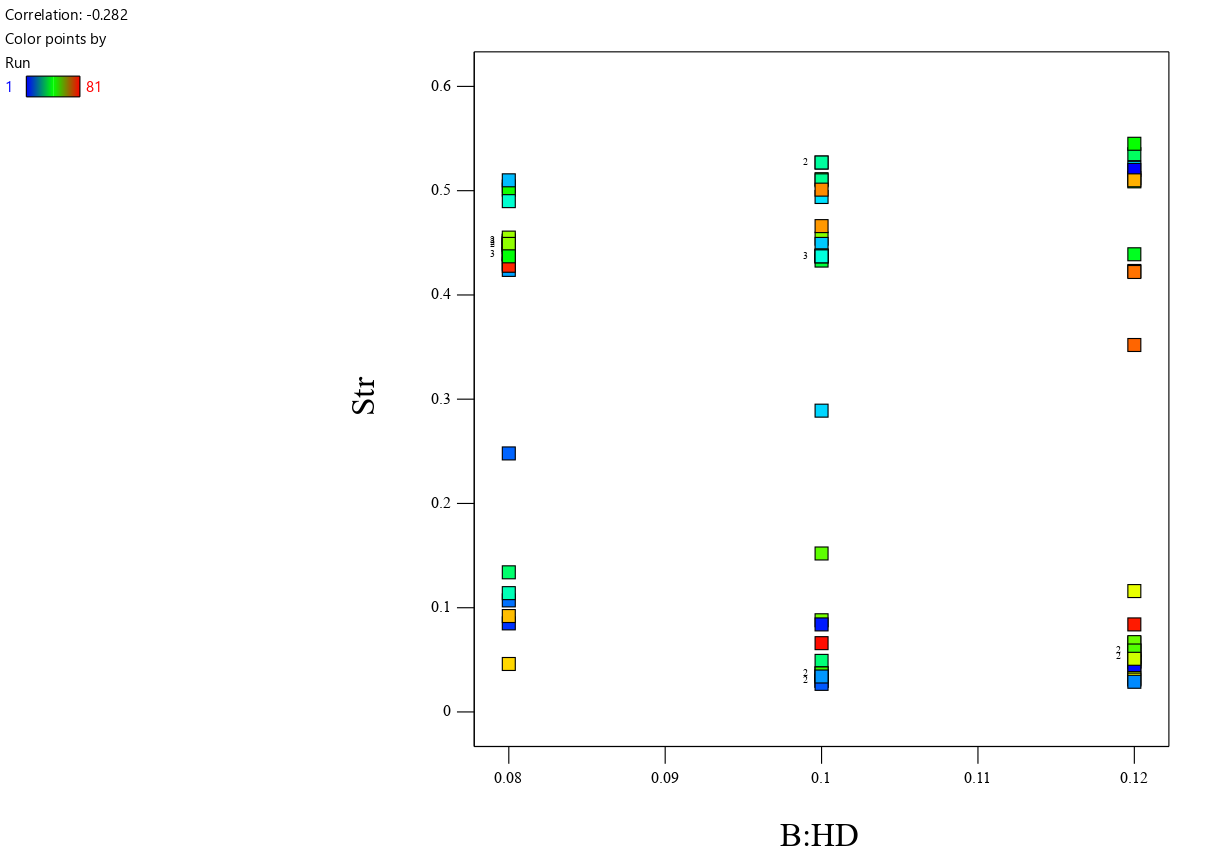


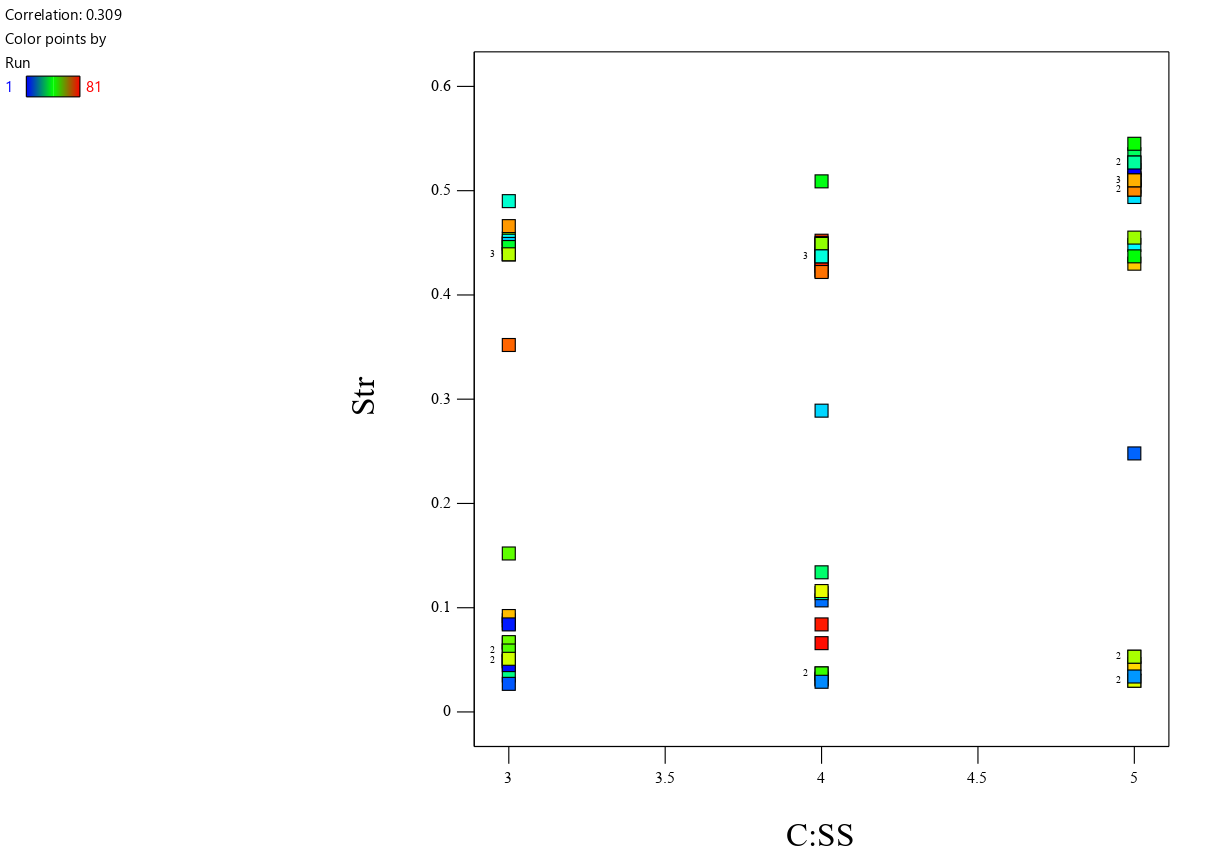


**Figure 5:** Scatter plots showing the correlation of texture aspect ratio (S_tr_) with laser process parameters such as laser power, hatch distance and scan speed.


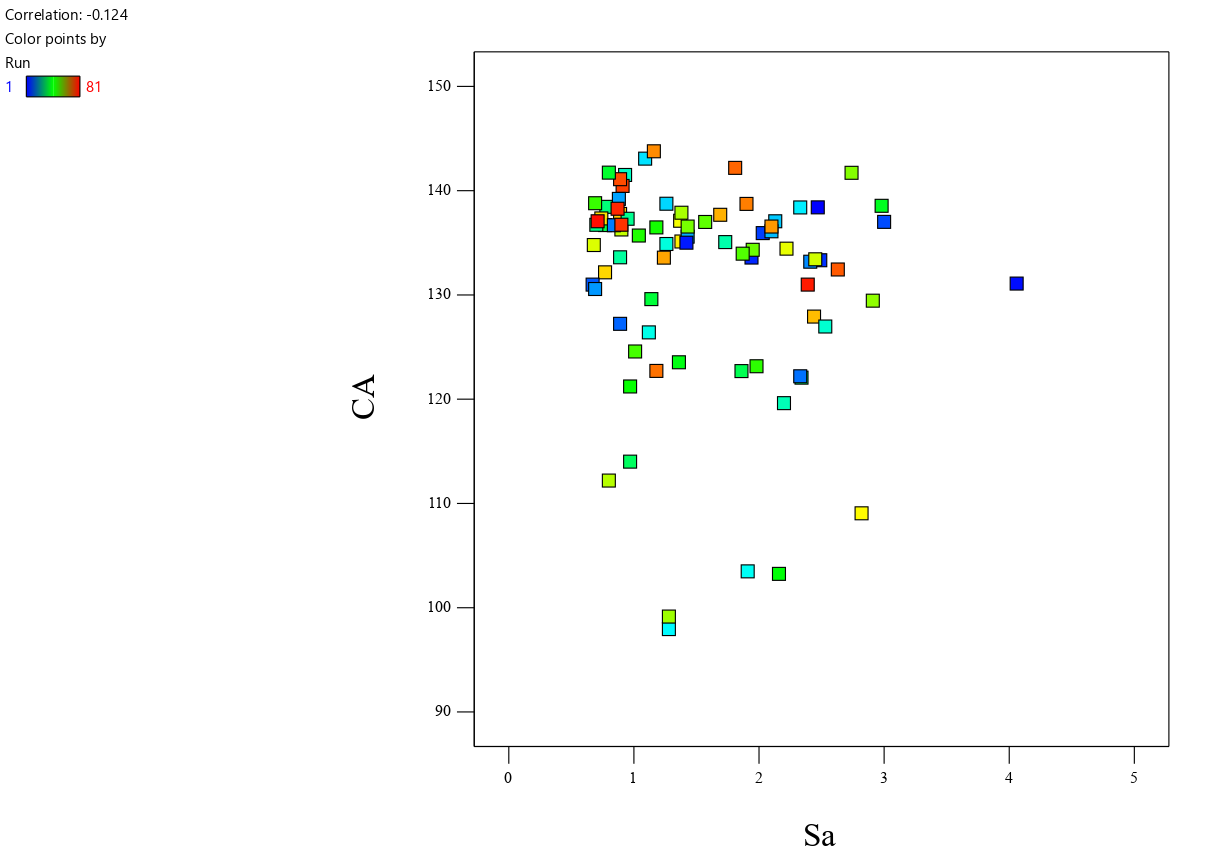

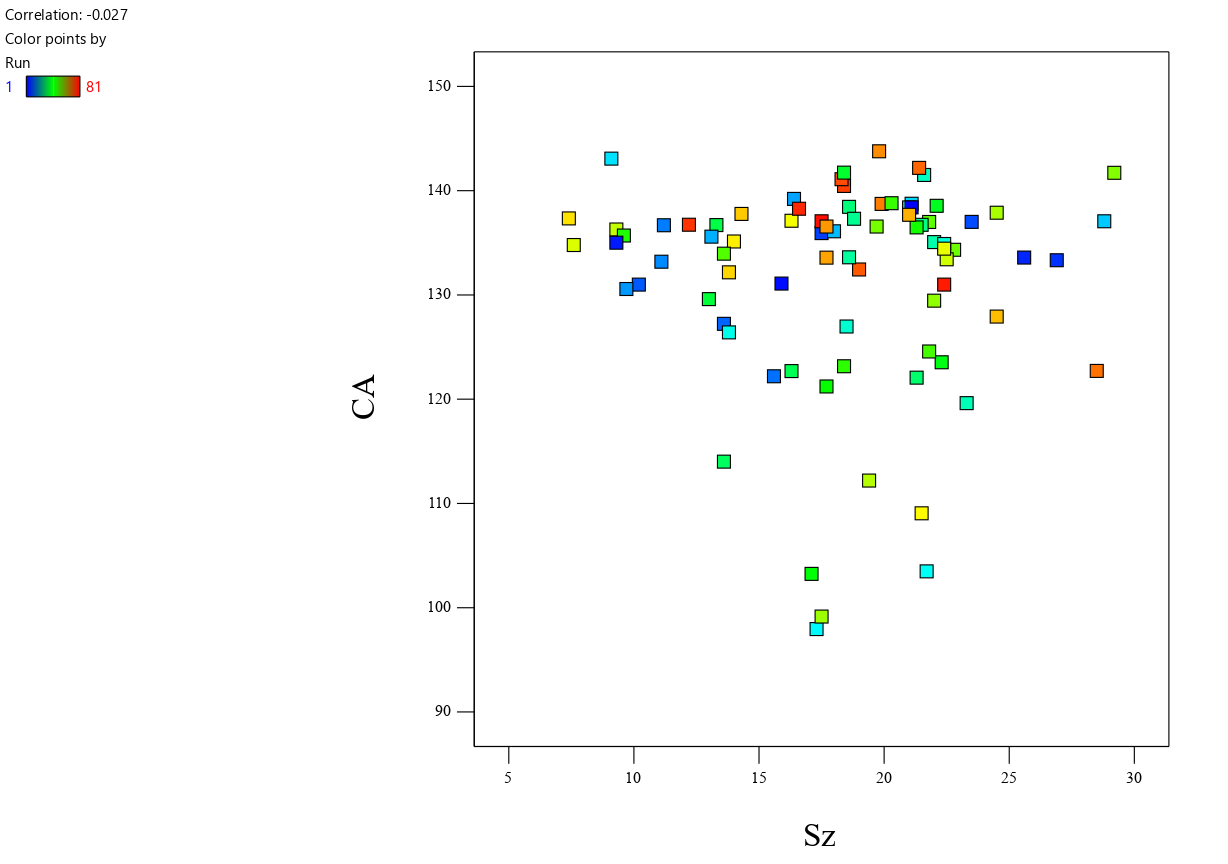


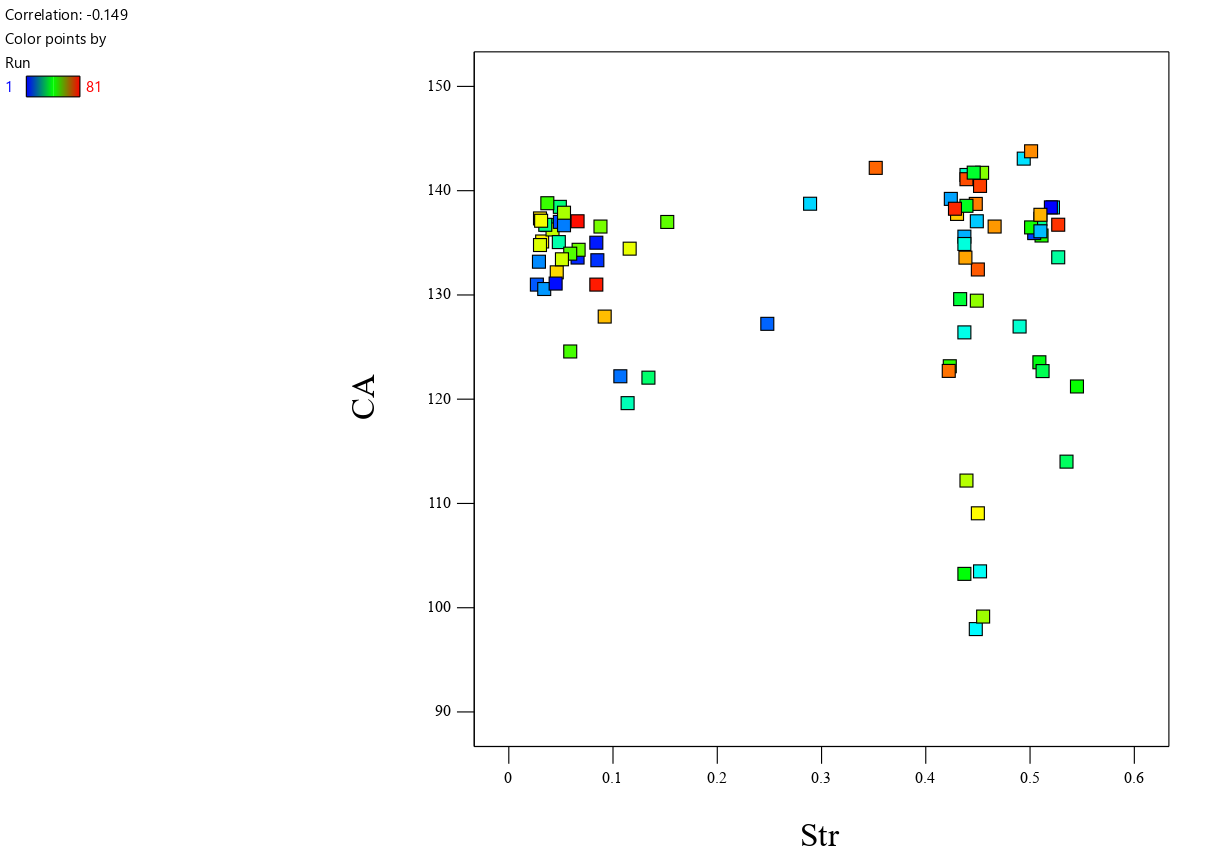


**Figure 5:** Scatter plots showing the correlation of water contact angle with other surface roughness attributes such as average surface roughness (S_a_), maximum depth surface roughness (S_z_) and texture aspect ratio (S_tr_)

# Analysis of variance

## ANOVA for Sa (Reduced Quartic model)

| **Source** | **Sum of Squares** | **df** | **Mean Square** | **F-value** | **p-value** |  |
| --- | --- | --- | --- | --- | --- | --- |
| **Model** | 2.55 | 12 | 0.2125 | 23.27 | < 0.0001 | **significant** |
| A-LP | 1.12 | 1 | 1.12 | 122.67 | < 0.0001 |  |
| B-HD | 0.0433 | 1 | 0.0433 | 4.75 | 0.0328 |  |
| C-SS | 0.2261 | 1 | 0.2261 | 24.75 | < 0.0001 |  |
| AB | 0.1119 | 1 | 0.1119 | 12.25 | 0.0008 |  |
| AC | 0.1066 | 1 | 0.1066 | 11.67 | 0.0011 |  |
| BC | 0.0266 | 1 | 0.0266 | 2.91 | 0.0923 |  |
| A² | 0.1015 | 1 | 0.1015 | 11.12 | 0.0014 |  |
| B² | 0.0477 | 1 | 0.0477 | 5.23 | 0.0253 |  |
| ABC | 0.0634 | 1 | 0.0634 | 6.94 | 0.0104 |  |
| A²B | 0.1784 | 1 | 0.1784 | 19.53 | < 0.0001 |  |
| A²B² | 0.0655 | 1 | 0.0655 | 7.18 | 0.0093 |  |
| AB²C | 0.1062 | 1 | 0.1062 | 11.62 | 0.0011 |  |
| **Residual** | 0.6210 | 68 | 0.0091 |  |  |  |
| Lack of Fit | 0.1136 | 14 | 0.0081 | 0.8639 | 0.6000 | not significant |
| Pure Error | 0.5074 | 54 | 0.0094 |  |  |  |
| **Cor Total** | 3.17 | 80 |  |  |  |  |

**Note**: The Model F-value of 23.27 implies the model is significant. There is only a 0.01% chance that an F-value this large could occur due to noise. The Lack of Fit F-value of 0.86 implies the Lack of Fit is not significant relative to the pure error. There is a 60.00% chance that a Lack of Fit F-value this large could occur due to noise. Non-significant lack of fit is good -- we want the model to fit.

## ANOVA for Sz (Reduced Quartic model)

| **Source** | **Sum of Squares** | **df** | **Mean Square** | **F-value** | **p-value** |  |
| --- | --- | --- | --- | --- | --- | --- |
| **Model** | 985.29 | 9 | 109.48 | 7.95 | < 0.0001 | **significant** |
| A-LP | 202.68 | 1 | 202.68 | 14.72 | 0.0003 |  |
| B-HD | 0.3200 | 1 | 0.3200 | 0.0232 | 0.8792 |  |
| C-SS | 351.65 | 1 | 351.65 | 25.55 | < 0.0001 |  |
| AB | 8.60 | 1 | 8.60 | 0.6251 | 0.4318 |  |
| A² | 54.40 | 1 | 54.40 | 3.95 | 0.0507 |  |
| B² | 166.43 | 1 | 166.43 | 12.09 | 0.0009 |  |
| A²B | 0.0181 | 1 | 0.0181 | 0.0013 | 0.9711 |  |
| AB² | 9.60 | 1 | 9.60 | 0.6975 | 0.4064 |  |
| A²B² | 53.78 | 1 | 53.78 | 3.91 | 0.0520 |  |
| **Residual** | 977.27 | 71 | 13.76 |  |  |  |
| Lack of Fit | 186.77 | 17 | 10.99 | 0.7505 | 0.7379 | not significant |
| Pure Error | 790.50 | 54 | 14.64 |  |  |  |
| **Cor Total** | 1962.56 | 80 |  |  |  |  |

Note: The Model F-value of 7.95 implies the model is significant. There is only a 0.01% chance that an F-value this large could occur due to noise. The Lack of Fit F-value of 0.75 implies the Lack of Fit is not significant relative to the pure error. There is a 73.79% chance that a Lack of Fit F-value this large could occur due to noise. Non-significant lack of fit is good -- we want the model to fit.

## ANOVA for Str (Reduced Sixth model)

| **Source** | **Sum of Squares** | **df** | **Mean Square** | **F-value** | **p-value** |  |
| --- | --- | --- | --- | --- | --- | --- |
| **Model** | 2.86 | 16 | 0.1789 | 27.99 | < 0.0001 | **significant** |
| A-LP | 0.2683 | 1 | 0.2683 | 41.98 | < 0.0001 |  |
| B-HD | 0.2596 | 1 | 0.2596 | 40.61 | < 0.0001 |  |
| C-SS | 0.2329 | 1 | 0.2329 | 36.43 | < 0.0001 |  |
| AB | 0.1865 | 1 | 0.1865 | 29.17 | < 0.0001 |  |
| BC | 0.1654 | 1 | 0.1654 | 25.88 | < 0.0001 |  |
| A² | 0.3459 | 1 | 0.3459 | 54.11 | < 0.0001 |  |
| C² | 0.0554 | 1 | 0.0554 | 8.66 | 0.0045 |  |
| A²C | 0.1327 | 1 | 0.1327 | 20.76 | < 0.0001 |  |
| AB² | 0.4090 | 1 | 0.4090 | 63.98 | < 0.0001 |  |
| AC² | 0.0697 | 1 | 0.0697 | 10.91 | 0.0016 |  |
| B²C | 0.0254 | 1 | 0.0254 | 3.98 | 0.0503 |  |
| A²BC | 0.0543 | 1 | 0.0543 | 8.50 | 0.0049 |  |
| A²C² | 0.1020 | 1 | 0.1020 | 15.96 | 0.0002 |  |
| AB²C | 0.4082 | 1 | 0.4082 | 63.85 | < 0.0001 |  |
| ABC² | 0.0556 | 1 | 0.0556 | 8.69 | 0.0045 |  |
| A²B²C | 0.0388 | 1 | 0.0388 | 6.07 | 0.0165 |  |
| **Residual** | 0.4091 | 64 | 0.0064 |  |  |  |
| Lack of Fit | 0.0624 | 10 | 0.0062 | 0.9720 | 0.4781 | not significant |
| Pure Error | 0.3467 | 54 | 0.0064 |  |  |  |
| **Cor Total** | 3.27 | 80 |  |  |  |  |

Note: The Model F-value of 27.99 implies the model is significant. There is only a 0.01% chance that an F-value this large could occur due to noise. The Lack of Fit F-value of 0.97 implies the Lack of Fit is not significant relative to the pure error. There is a 47.81% chance that a Lack of Fit F-value this large could occur due to noise. Non-significant lack of fit is good -- we want the model to fit.

## ANOVA for Contact angle (Reduced sixth model)

| **Source** | **Sum of Squares** | **df** | **Mean Square** | **F-value** | **p-value** |  |
| --- | --- | --- | --- | --- | --- | --- |
| **Model** | 7210.14 | 20 | 360.51 | 33.99 | < 0.0001 | **significant** |
| B-HD | 162.34 | 1 | 162.34 | 15.31 | 0.0002 |  |
| C-SS | 32.62 | 1 | 32.62 | 3.08 | 0.0846 |  |
| AB | 230.48 | 1 | 230.48 | 21.73 | < 0.0001 |  |
| A² | 142.69 | 1 | 142.69 | 13.45 | 0.0005 |  |
| B² | 70.04 | 1 | 70.04 | 6.60 | 0.0127 |  |
| C² | 146.27 | 1 | 146.27 | 13.79 | 0.0004 |  |
| ABC | 67.50 | 1 | 67.50 | 6.36 | 0.0143 |  |
| A²B | 225.05 | 1 | 225.05 | 21.22 | < 0.0001 |  |
| A²C | 42.55 | 1 | 42.55 | 4.01 | 0.0497 |  |
| AB² | 267.77 | 1 | 267.77 | 25.25 | < 0.0001 |  |
| AC² | 92.03 | 1 | 92.03 | 8.68 | 0.0046 |  |
| B²C | 208.22 | 1 | 208.22 | 19.63 | < 0.0001 |  |
| BC² | 975.31 | 1 | 975.31 | 91.96 | < 0.0001 |  |
| A²C² | 131.72 | 1 | 131.72 | 12.42 | 0.0008 |  |
| AB²C | 124.53 | 1 | 124.53 | 11.74 | 0.0011 |  |
| ABC² | 89.76 | 1 | 89.76 | 8.46 | 0.0051 |  |
| B²C² | 1009.42 | 1 | 1009.42 | 95.17 | < 0.0001 |  |
| A²B²C | 186.73 | 1 | 186.73 | 17.61 | < 0.0001 |  |
| A²BC² | 777.29 | 1 | 777.29 | 73.29 | < 0.0001 |  |
| A²B²C² | 1489.16 | 1 | 1489.16 | 140.41 | < 0.0001 |  |
| **Residual** | 636.37 | 60 | 10.61 |  |  |  |
| Lack of Fit | 61.99 | 6 | 10.33 | 0.9714 | 0.4534 | not significant |
| Pure Error | 574.37 | 54 | 10.64 |  |  |  |
| **Cor Total** | 7846.51 | 80 |  |  |  |  |

Note: The Model F-value of 33.99 implies the model is significant. There is only a 0.01% chance that an F-value this large could occur due to noise. The Lack of Fit F-value of 0.97 implies the Lack of Fit is not significant relative to the pure error. There is a 45.34% chance that a Lack of Fit F-value this large could occur due to noise. Non-significant lack of fit is good -- we want the model to fit.

## Model Significance factors

Table. 4. ANOVA significance parameters for RSM analysis

| **Response** | **p-value** | |
| --- | --- | --- |
|  | **Model** | **Lack of fit** |
| Arithmetic mean height (Sa) | < 0.0001 | 0.85 |
| Maximum height (Sz) | < 0.0001 | 0.08 |
| Texture aspect ratio (Str) | < 0.0001 | 0.89 |
| Contact Angle (CA) | < 0.0001 | 0.45 |

**xNote: If the p value = <0.05 is significant; the p value > 0.05 is non significant**

Table. 5. ANOVA significance adjusted and predicted R^2^ parameters for RSM analysis

| **Response** |  |  |
| --- | --- | --- |
|  | **Adjusted R^2^** | **Predicted R^2^** |
| Arithmetic mean height (Sa) | 0.7695 | 0.7168 |
| Maximum height (Sz) | 0.6602 | 0.5611 |
| Texture aspect ratio (Str) | 0.8436 | 0.7889 |
| Contact Angle (CA) | 0.8919 | 0.8513 |

# Results of height roughness (Sa), (Sz) with varying laser process parameter

## Effect on arithmatic mean roughness (Sa)

**
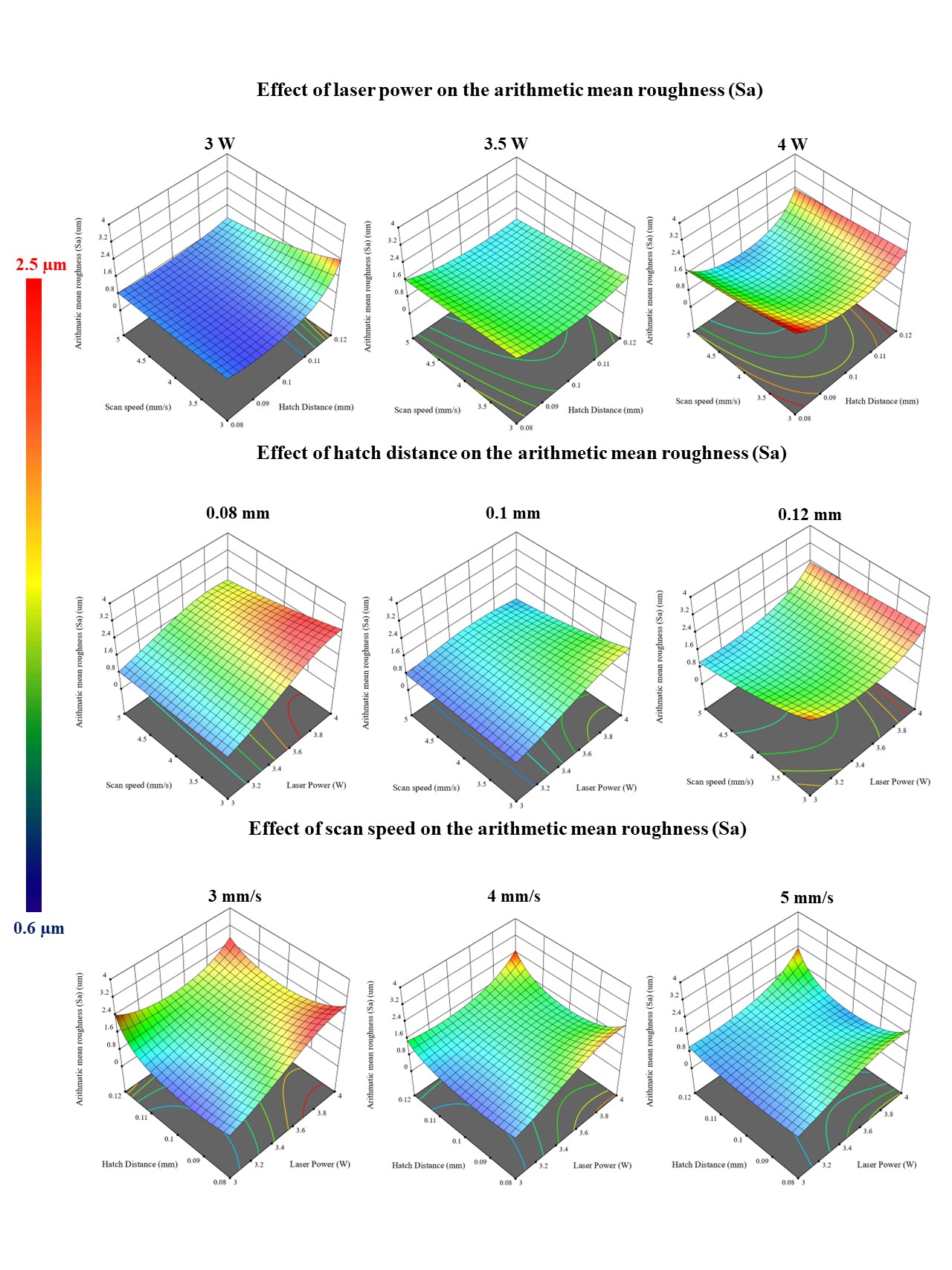
**

**Figure 6** The response surface method (RSM) graphs showing the effect of the input processing parameter such as laser power, hatch distance and scan speed on the output measured arithmetic surface roughness (Sa) values

## Effect on maximum height roughness (Sz)

**
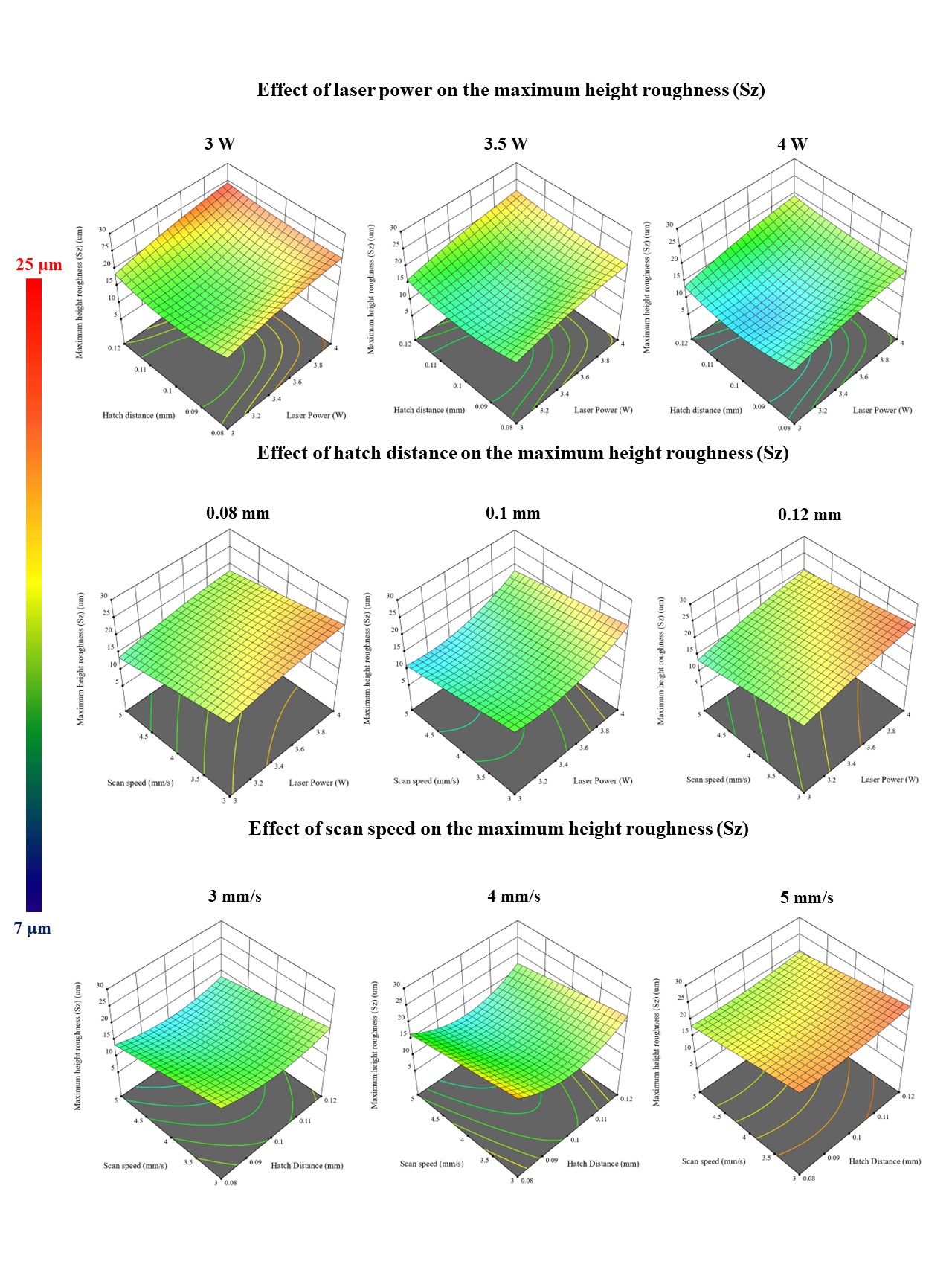
**

**Figure 7** The response surface method (RSM) graphs showing the effect of the input processing parameter such as laser power, hatch distance and scan speed on the output measured maximum height surface roughness (Sz) values

## Model significance parameters of Sa and Sz data.


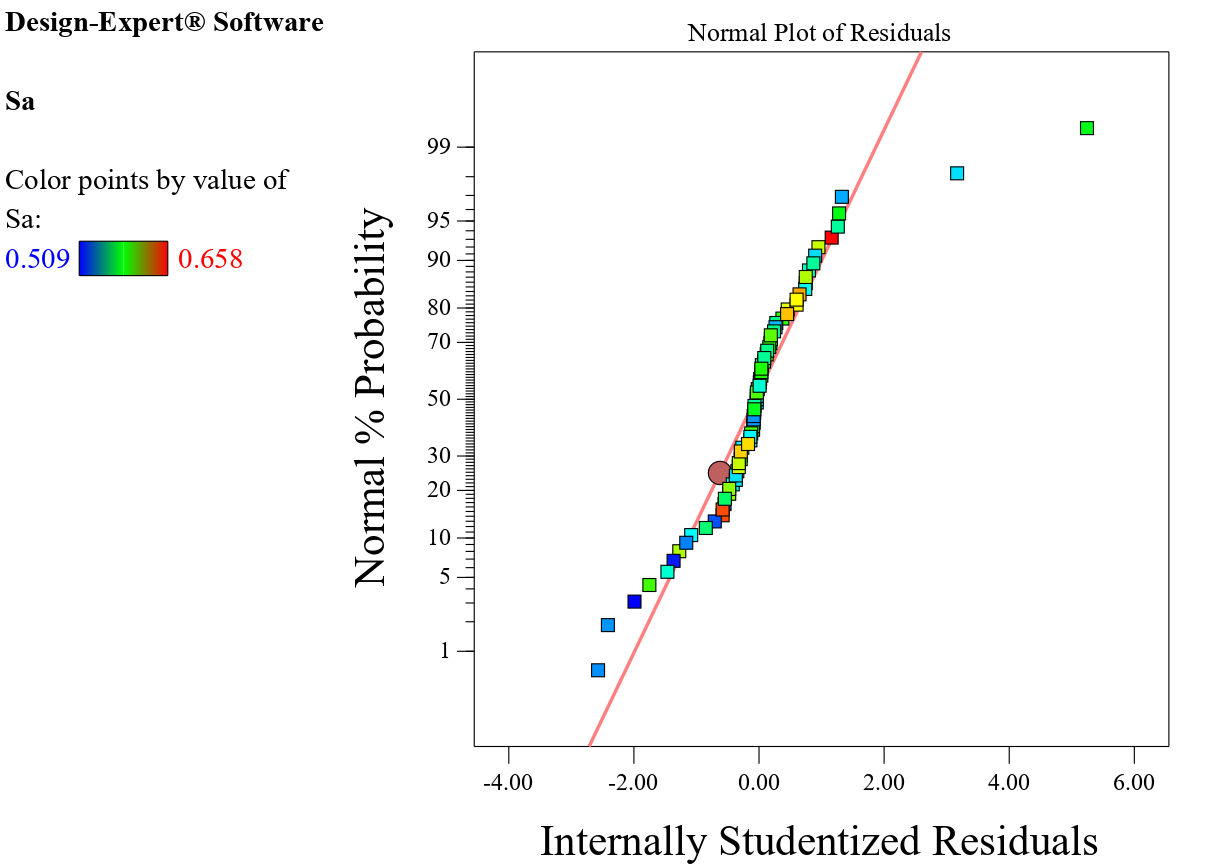

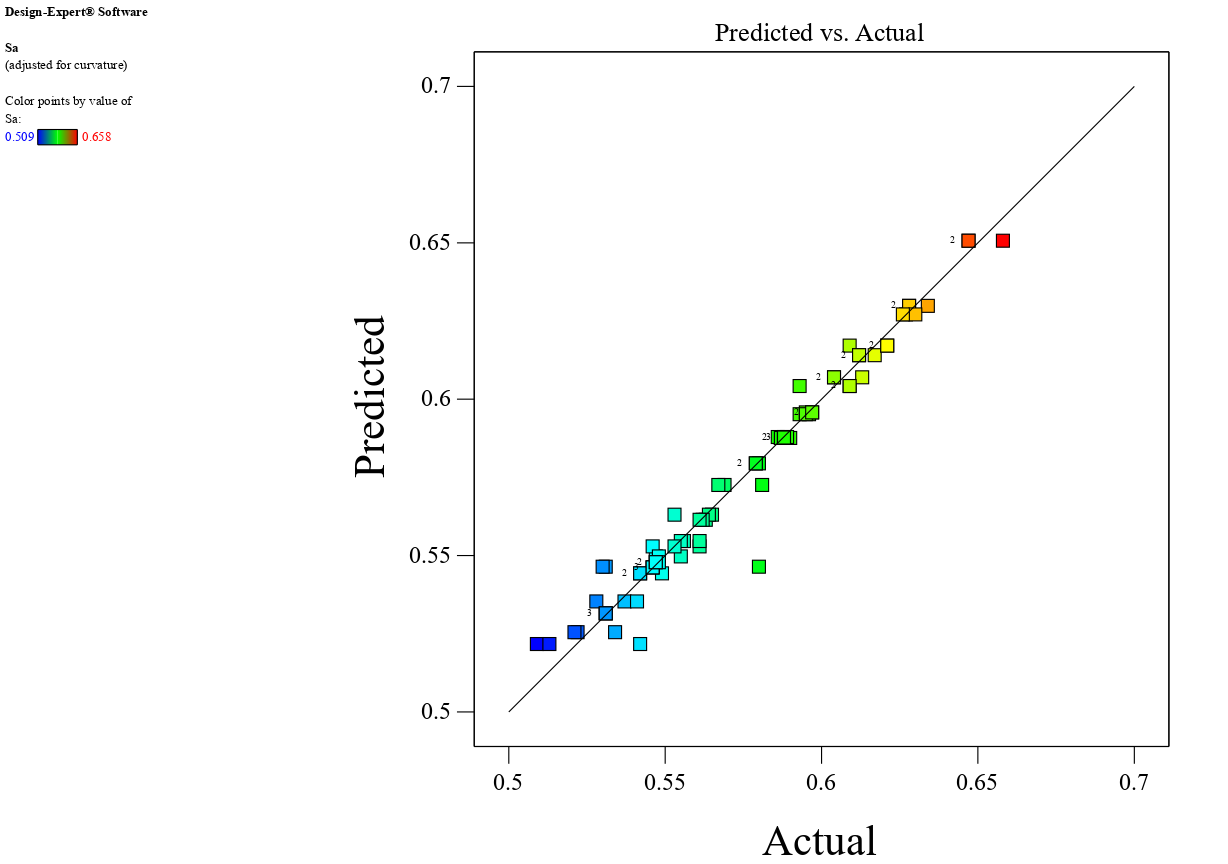


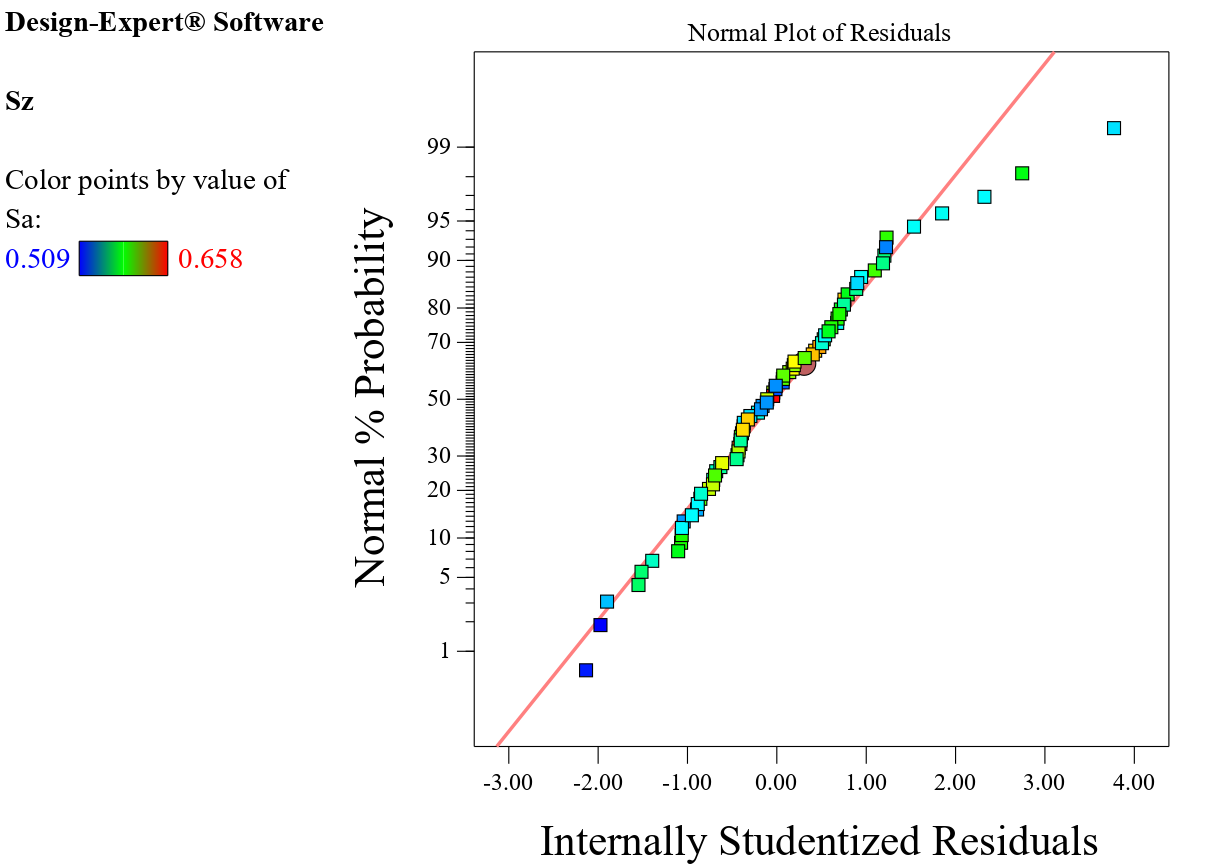

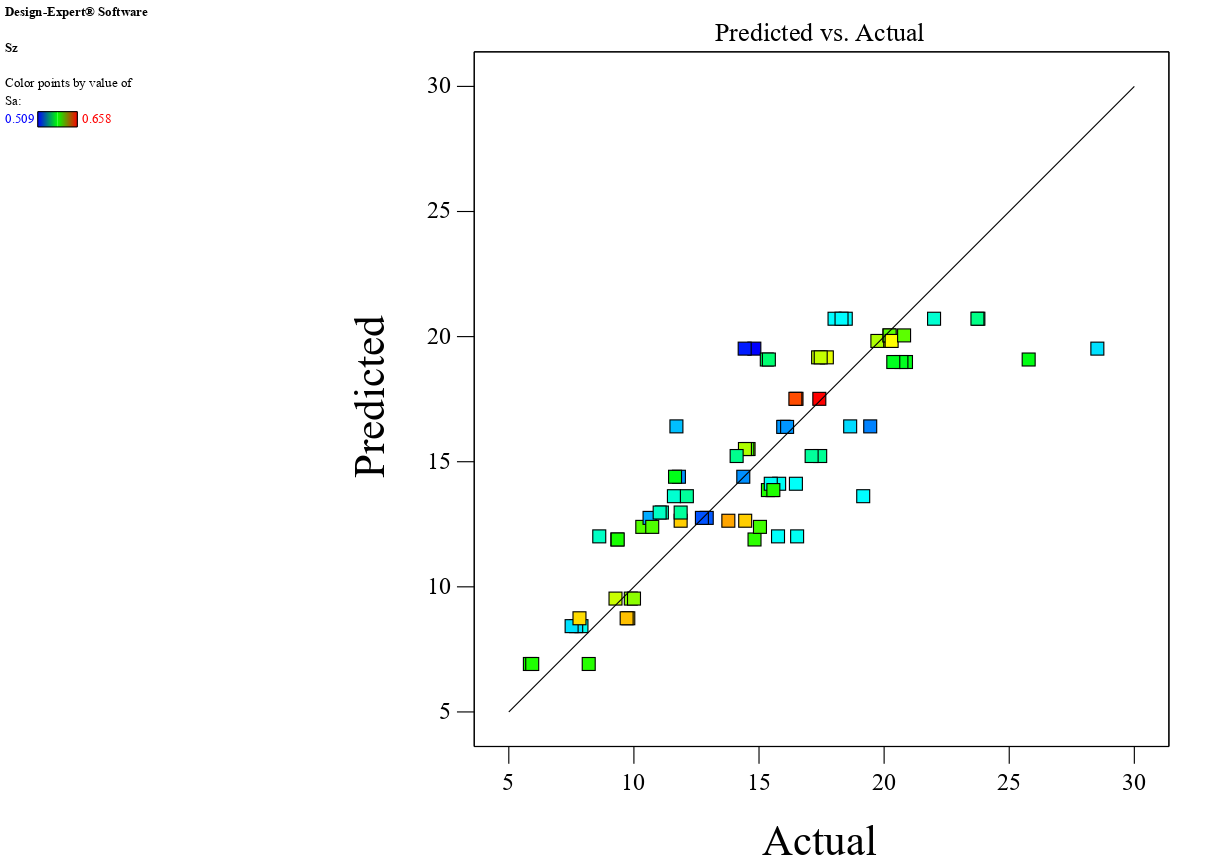


**Figure 8** Normal probability with internally studentized residuals for (Sa) and (Sz) models and predicted vs actual curve for arithmetic mean roughness (Sa) and (Sz)model derived.

# Results of spatial roughness (Str) with varying laser process parameter

## Effects on texture aspect ratio (Str)


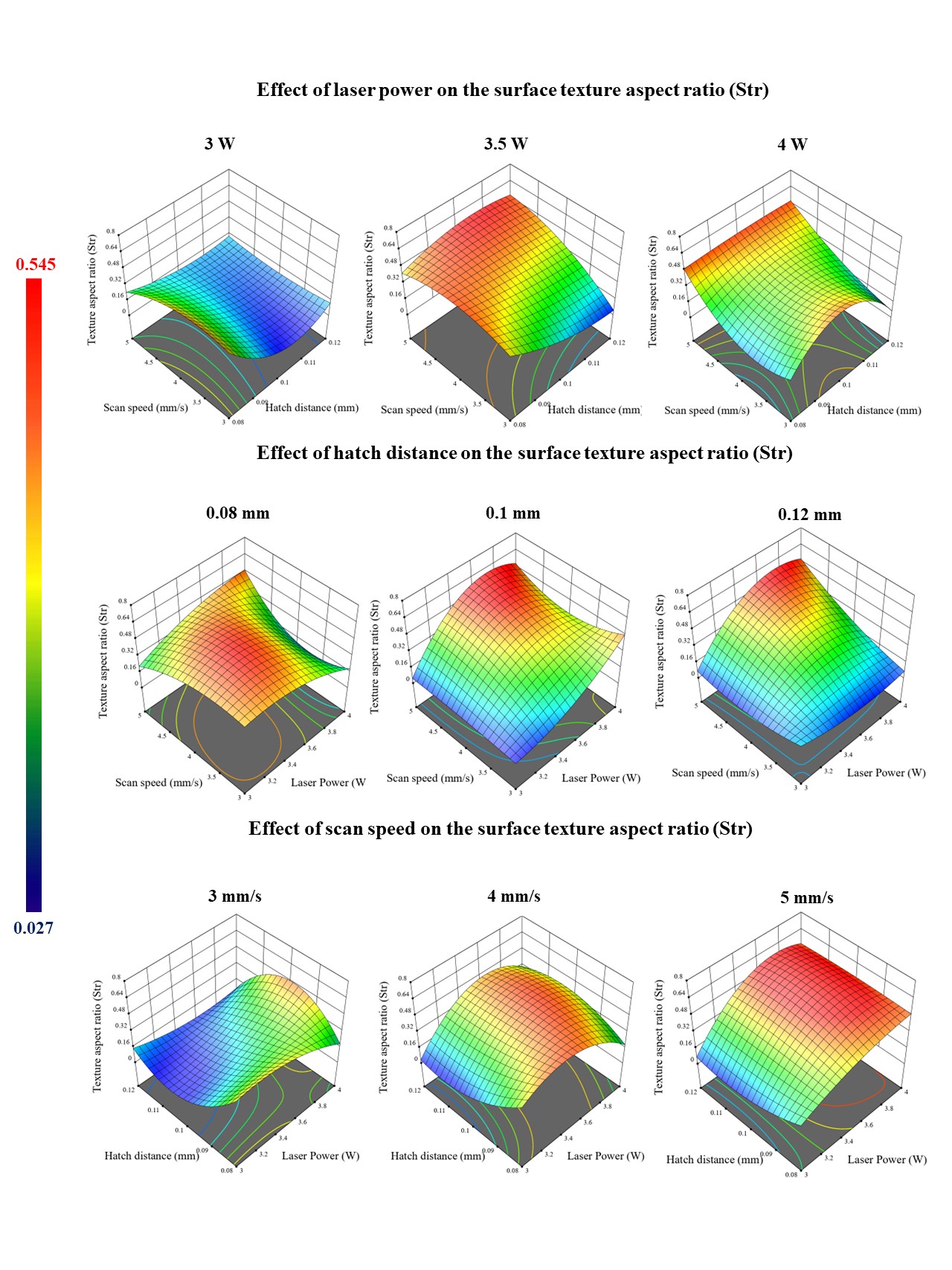


**Figure 9** The response surface method (RSM) graphs showing the effect of the input processing parameter such as laser power, hatch distance and scan speed on the output measured texture aspect ratio (Str) values

## Model Significance parameters for Str


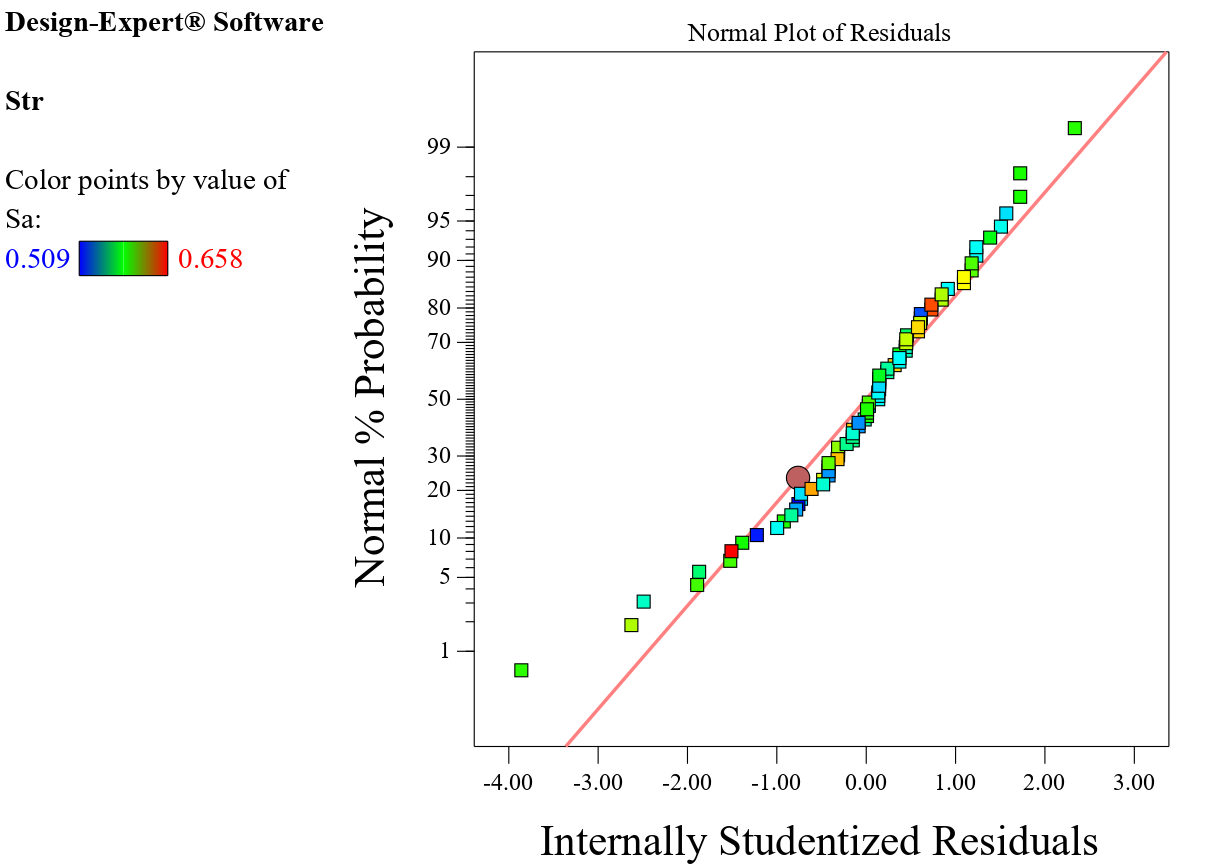

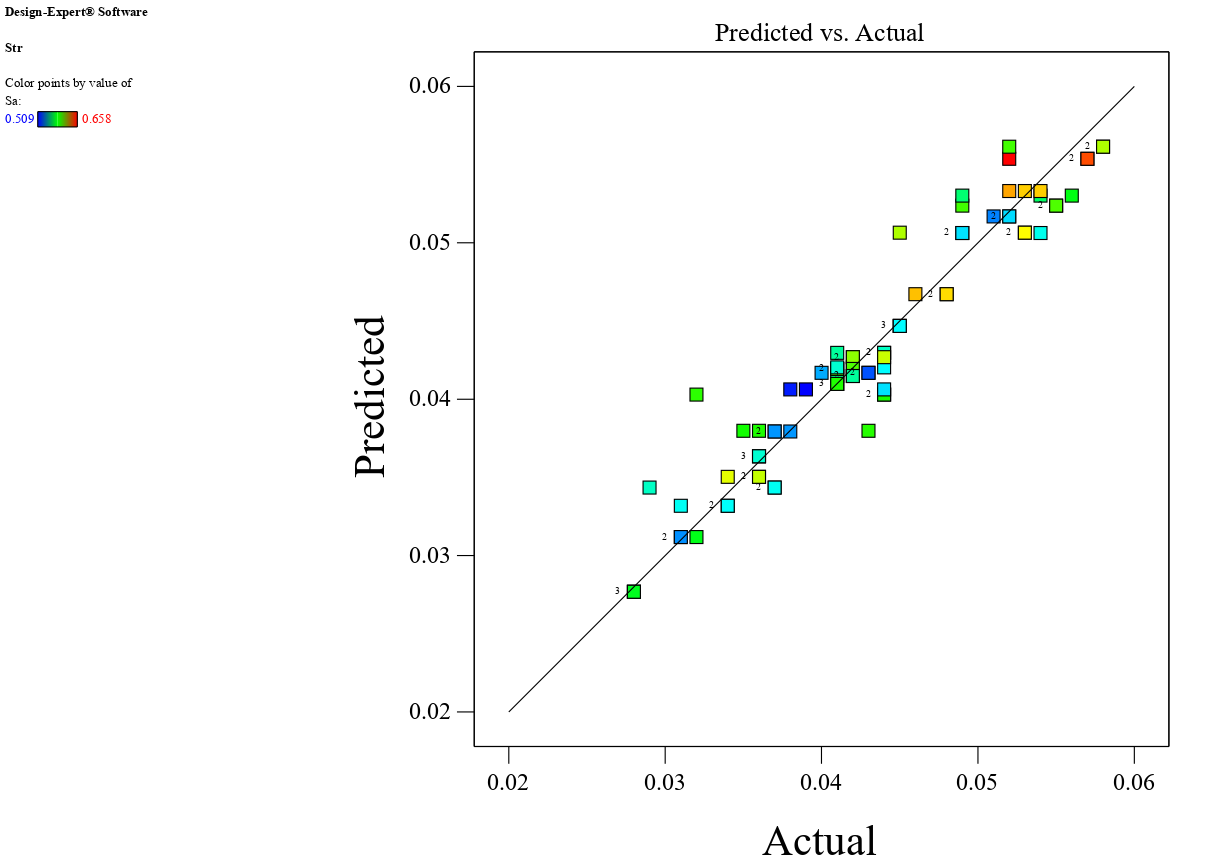


**Figure 10** Normal probability with internally studentized residuals for (Str) model and predicted vs actual curve for arithmetic mean roughness (Str) model derived.

# Results of contact angle with varying laser process parameter

## Effect on contact angle

**Figure 11** Mean Effect plot of water contact angle showing the effect of the input processing parameter such as LP- laser power, HD- hatch distance and SS- scan speed.


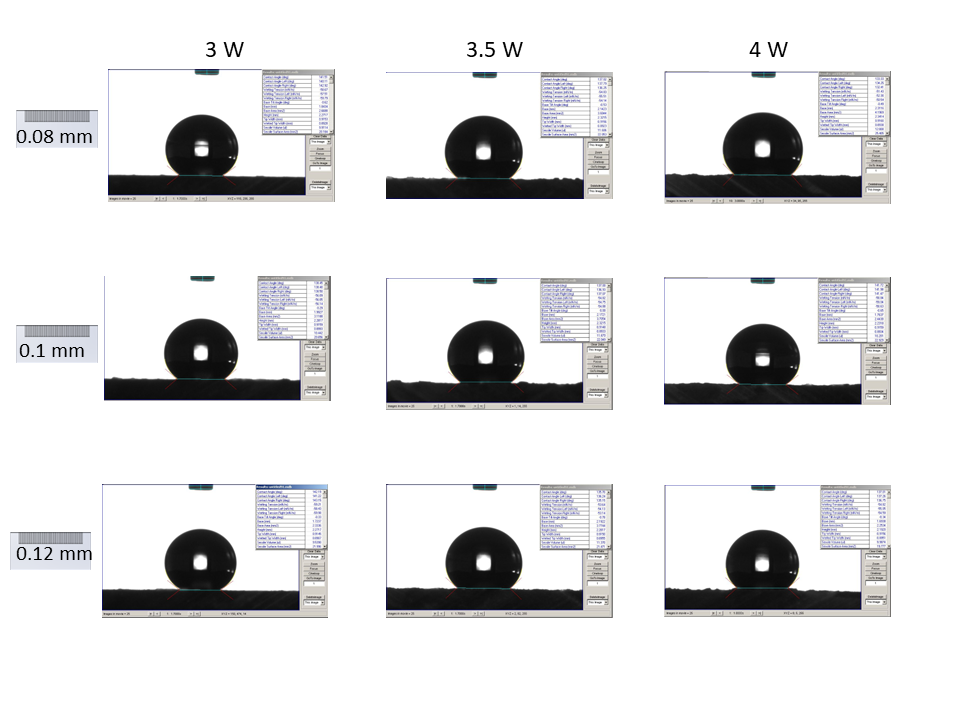


**Figure 12** Contact angle measurements with different powers and hatch speeds with 3 mm/s scan speed

**
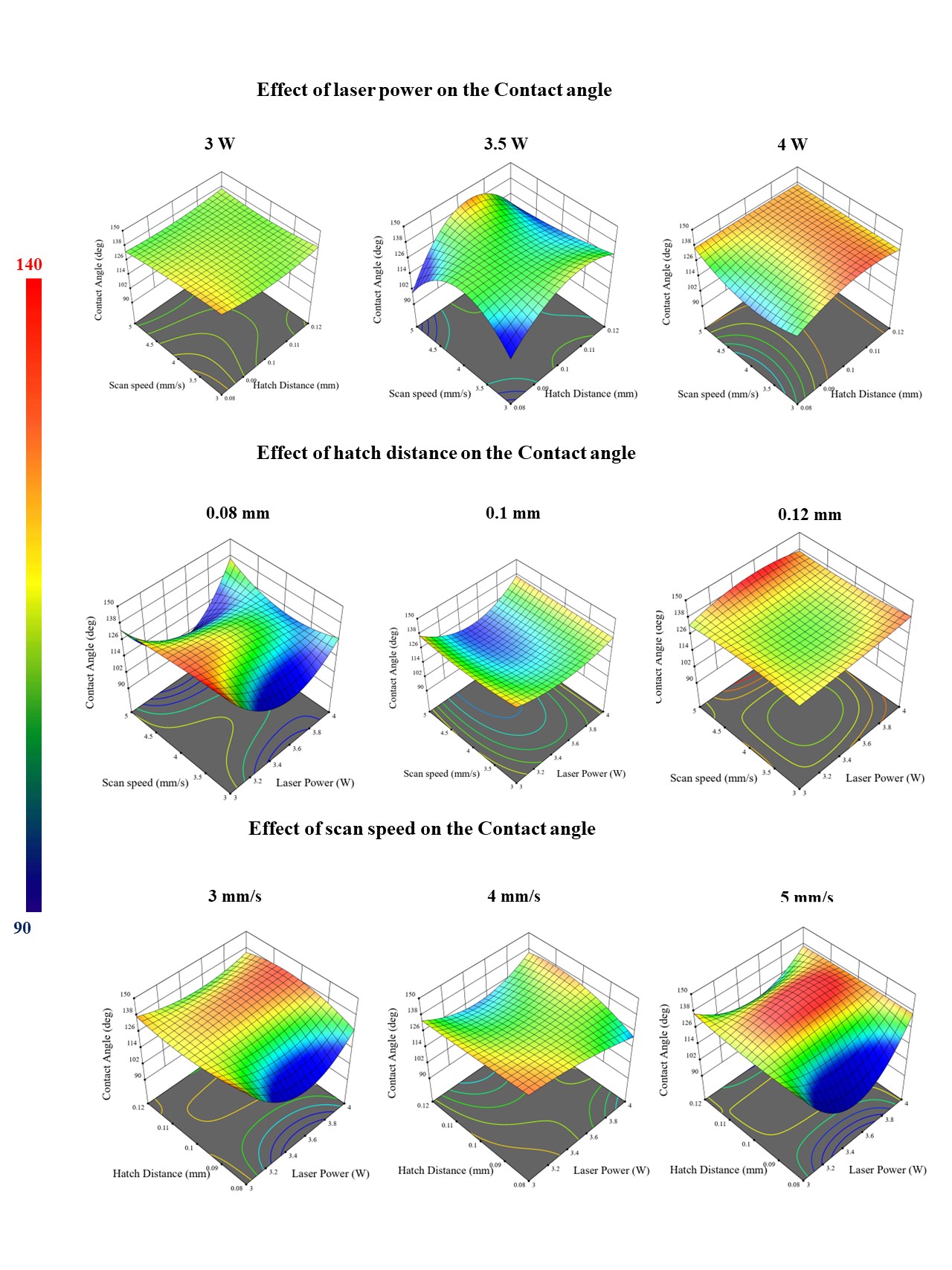
**

**Figure 13** The response surface method (RSM) graphs showing the effect of the input processing parameter such as laser power, hatch distance and scan speed on the output measured water contact angle values

## Model significance parameters of contact angle


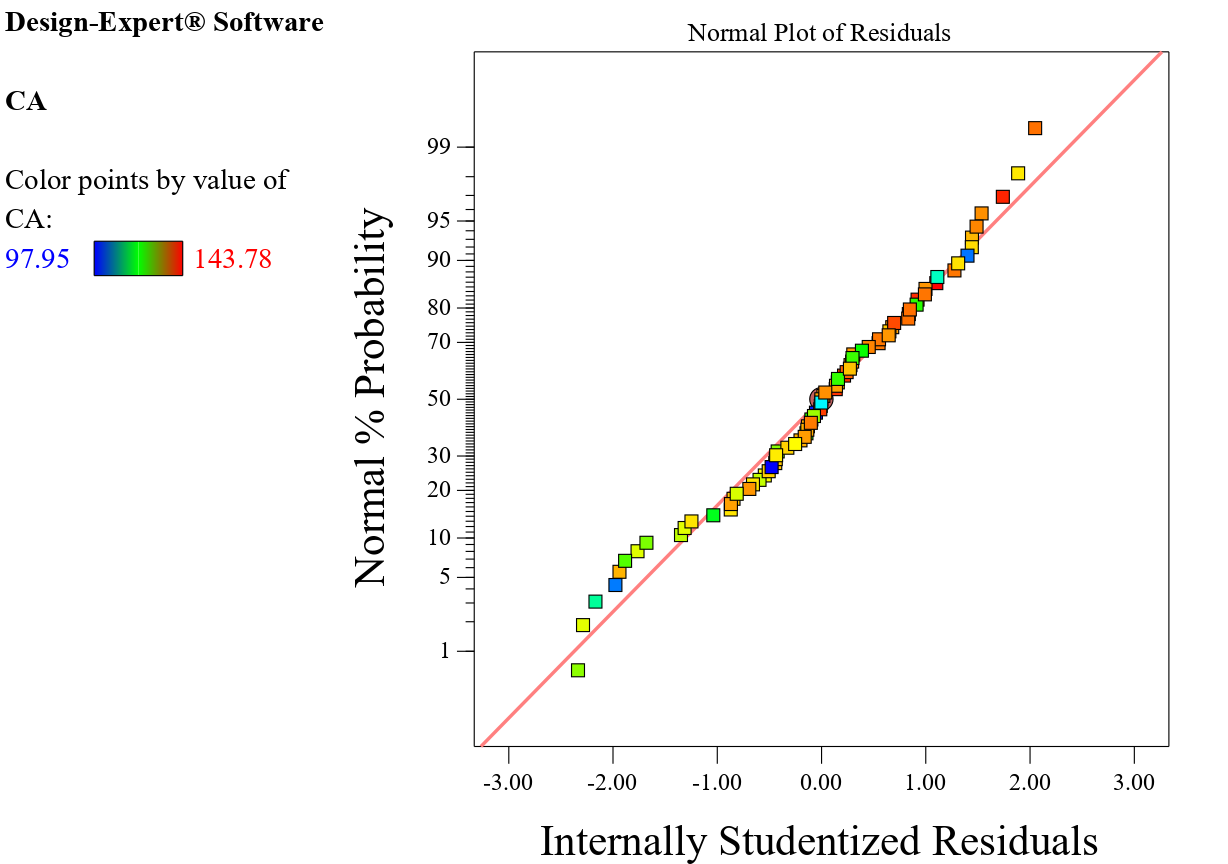

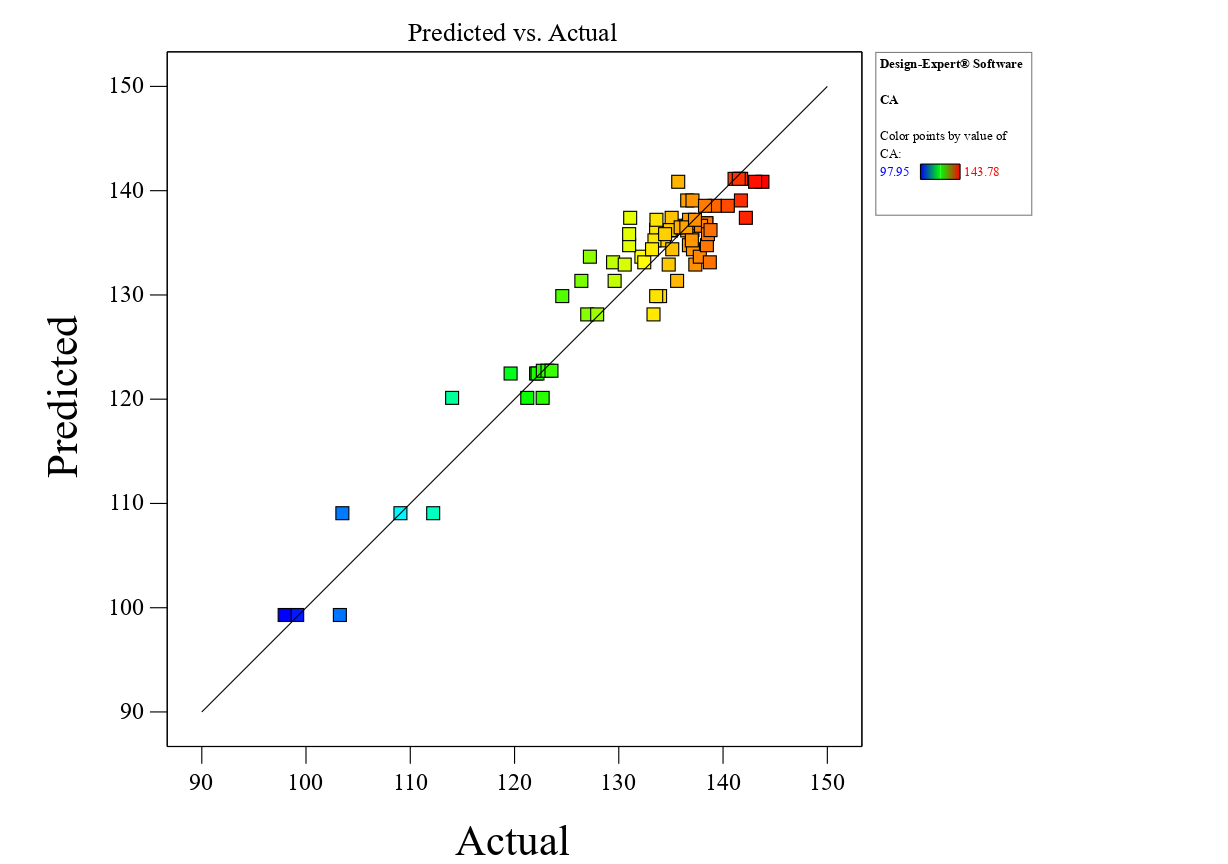


**Figure 14** Normal plot and predicted vs actual results for contact angle measurements.

# Results of numerical optimization

**Table 1** Solutions as obtained by Design-Expert based on the criterion. **(Note: The three highlighted solutions were validated)**

| **Number** | **LP** | **HD** | **SS** | **CA** | **Sa** | **Sz** | **Str** | **Desirability** |  |
| --- | --- | --- | --- | --- | --- | --- | --- | --- | --- |
| **1** | **3.577** | **0.103** | **5.000** | **141.878** | **0.644** | **17.881** | **0.054** | **0.979** | **Selected** |
| 2 | 3.580 | 0.103 | 5.000 | 141.875 | 0.644 | 17.893 | 0.054 | 0.979 |  |
| 3 | 3.592 | 0.103 | 5.000 | 141.872 | 0.643 | 17.917 | 0.054 | 0.979 |  |
| 4 | 3.590 | 0.103 | 5.000 | 141.865 | 0.644 | 17.919 | 0.054 | 0.979 |  |
| 5 | 3.572 | 0.104 | 5.000 | 141.863 | 0.643 | 17.855 | 0.053 | 0.979 |  |
| 6 | 3.599 | 0.103 | 5.000 | 141.863 | 0.642 | 17.927 | 0.053 | 0.979 |  |
| 7 | 3.542 | 0.103 | 5.000 | 141.845 | 0.645 | 17.752 | 0.054 | 0.979 |  |
| 8 | 3.593 | 0.103 | 5.000 | 141.844 | 0.644 | 17.928 | 0.054 | 0.979 |  |
| 9 | 3.570 | 0.103 | 5.000 | 141.843 | 0.645 | 17.870 | 0.054 | 0.979 |  |
| 10 | 3.610 | 0.104 | 5.000 | 141.833 | 0.641 | 17.935 | 0.053 | 0.979 |  |
| 11 | 3.566 | 0.104 | 5.000 | 141.822 | 0.642 | 17.823 | 0.053 | 0.978 |  |
| 12 | 3.583 | 0.103 | 4.999 | 141.847 | 0.643 | 17.887 | 0.054 | 0.978 |  |
| 13 | 3.527 | 0.103 | 5.000 | 141.813 | 0.645 | 17.677 | 0.054 | 0.978 |  |
| 14 | 3.611 | 0.103 | 5.000 | 141.813 | 0.643 | 17.955 | 0.054 | 0.978 |  |
| 15 | 3.570 | 0.105 | 5.000 | 141.757 | 0.641 | 17.822 | 0.053 | 0.978 |  |
| 16 | 3.605 | 0.102 | 5.000 | 141.698 | 0.645 | 17.952 | 0.054 | 0.977 |  |
| 17 | 3.492 | 0.103 | 5.000 | 141.689 | 0.645 | 17.454 | 0.054 | 0.977 |  |
| 18 | 3.470 | 0.103 | 5.000 | 141.552 | 0.646 | 17.290 | 0.054 | 0.975 |  |
| 19 | 3.457 | 0.105 | 5.000 | 141.346 | 0.642 | 17.142 | 0.053 | 0.973 |  |
| 20 | 3.704 | 0.105 | 5.000 | 141.292 | 0.632 | 17.812 | 0.051 | 0.972 |  |
| 21 | 3.593 | 0.103 | 4.983 | 141.527 | 0.642 | 17.821 | 0.053 | 0.971 |  |
| 22 | 3.749 | 0.103 | 5.000 | 141.111 | 0.631 | 17.600 | 0.051 | 0.970 |  |
| 23 | 3.406 | 0.104 | 5.000 | 141.064 | 0.642 | 16.644 | 0.053 | 0.970 |  |
| 24 | 3.686 | 0.100 | 5.000 | 140.638 | 0.642 | 17.804 | 0.054 | 0.965 |  |
| 25 | 3.818 | 0.102 | 5.000 | 140.345 | 0.622 | 17.018 | 0.049 | 0.962 |  |
| 26 | 3.744 | 0.107 | 5.000 | 140.134 | 0.622 | 17.615 | 0.048 | 0.959 |  |
| 27 | 3.832 | 0.104 | 5.000 | 140.129 | 0.618 | 16.979 | 0.047 | 0.959 |  |
| 28 | 3.835 | 0.105 | 5.000 | 140.016 | 0.616 | 16.993 | 0.047 | 0.958 |  |
| 29 | 3.833 | 0.101 | 5.000 | 139.932 | 0.622 | 16.743 | 0.049 | 0.957 |  |
| 30 | 3.890 | 0.103 | 5.000 | 139.329 | 0.610 | 16.218 | 0.045 | 0.950 |  |
| 31 | 3.890 | 0.105 | 5.000 | 139.219 | 0.608 | 16.409 | 0.044 | 0.949 |  |
| 32 | 3.877 | 0.100 | 5.000 | 139.191 | 0.615 | 16.097 | 0.047 | 0.949 |  |
| 33 | 3.914 | 0.101 | 5.000 | 138.843 | 0.607 | 15.680 | 0.045 | 0.945 |  |
| 34 | 3.923 | 0.102 | 5.000 | 138.748 | 0.604 | 15.665 | 0.044 | 0.944 |  |
| 35 | 3.955 | 0.105 | 5.000 | 138.117 | 0.597 | 15.559 | 0.041 | 0.936 |  |
| 36 | 3.961 | 0.101 | 5.000 | 137.971 | 0.597 | 14.802 | 0.043 | 0.934 |  |
| 37 | 3.990 | 0.102 | 5.000 | 137.443 | 0.590 | 14.484 | 0.041 | 0.928 |  |
| 38 | 3.987 | 0.108 | 5.000 | 137.398 | 0.592 | 15.624 | 0.039 | 0.928 |  |
| 39 | 4.000 | 0.103 | 5.000 | 137.224 | 0.587 | 14.499 | 0.039 | 0.926 |  |
| 40 | 4.000 | 0.104 | 5.000 | 137.224 | 0.588 | 14.581 | 0.039 | 0.926 |  |
| 41 | 4.000 | 0.111 | 5.000 | 137.153 | 0.594 | 16.317 | 0.037 | 0.925 |  |
| 42 | 4.000 | 0.113 | 5.000 | 137.120 | 0.597 | 16.763 | 0.036 | 0.924 |  |
| 43 | 4.000 | 0.115 | 5.000 | 137.047 | 0.602 | 17.598 | 0.036 | 0.924 |  |
| 44 | 4.000 | 0.117 | 5.000 | 136.986 | 0.606 | 18.212 | 0.035 | 0.923 |  |
| 45 | 4.000 | 0.119 | 5.000 | 136.920 | 0.611 | 18.822 | 0.035 | 0.922 |  |
| 46 | 4.000 | 0.088 | 5.000 | 136.908 | 0.604 | 12.653 | 0.047 | 0.922 |  |
| 47 | 4.000 | 0.086 | 5.000 | 136.806 | 0.610 | 12.580 | 0.049 | 0.921 |  |
| 48 | 3.998 | 0.120 | 5.000 | 136.740 | 0.614 | 19.144 | 0.035 | 0.920 |  |
| 49 | 4.000 | 0.084 | 5.000 | 136.700 | 0.617 | 12.562 | 0.051 | 0.920 |  |
| 50 | 3.000 | 0.120 | 5.000 | 136.641 | 0.617 | 19.829 | 0.051 | 0.919 |  |
| 51 | 3.001 | 0.119 | 5.000 | 136.290 | 0.616 | 18.622 | 0.050 | 0.915 |  |
| 52 | 3.110 | 0.104 | 5.000 | 136.252 | 0.614 | 11.191 | 0.045 | 0.914 |  |
| 53 | 3.000 | 0.120 | 4.967 | 136.482 | 0.619 | 19.147 | 0.051 | 0.909 |  |
| 54 | 3.090 | 0.110 | 5.000 | 135.395 | 0.611 | 12.459 | 0.045 | 0.904 |  |
| 55 | 3.065 | 0.109 | 5.000 | 135.107 | 0.609 | 11.757 | 0.044 | 0.900 |  |
| 56 | 3.094 | 0.112 | 5.000 | 135.001 | 0.610 | 13.327 | 0.046 | 0.899 |  |
| 57 | 3.026 | 0.114 | 5.000 | 134.769 | 0.608 | 13.877 | 0.045 | 0.896 |  |
| 58 | 3.041 | 0.110 | 5.000 | 134.748 | 0.607 | 11.907 | 0.044 | 0.896 |  |
| 59 | 3.032 | 0.113 | 5.000 | 134.717 | 0.608 | 13.773 | 0.045 | 0.896 |  |
| 60 | 3.000 | 0.112 | 5.000 | 134.663 | 0.606 | 12.490 | 0.044 | 0.895 |  |
| 61 | 3.000 | 0.080 | 5.000 | 133.670 | 0.553 | 13.624 | 0.042 | 0.883 |  |
| 62 | 3.000 | 0.080 | 4.985 | 133.757 | 0.553 | 13.697 | 0.042 | 0.881 |  |
| 63 | 3.000 | 0.080 | 4.967 | 133.866 | 0.552 | 13.786 | 0.042 | 0.878 |  |
| 64 | 3.022 | 0.096 | 5.000 | 133.057 | 0.588 | 7.444 | 0.039 | 0.875 |  |
| 65 | 3.000 | 0.087 | 5.000 | 132.888 | 0.566 | 8.991 | 0.039 | 0.873 |  |
| 66 | 3.000 | 0.088 | 5.000 | 132.808 | 0.568 | 8.428 | 0.038 | 0.872 |  |
| 67 | 3.000 | 0.089 | 5.000 | 132.797 | 0.569 | 8.343 | 0.038 | 0.872 |  |
| 68 | 3.000 | 0.090 | 5.000 | 132.744 | 0.571 | 7.922 | 0.038 | 0.871 |  |
| 69 | 3.000 | 0.092 | 5.000 | 132.685 | 0.574 | 7.326 | 0.038 | 0.871 |  |


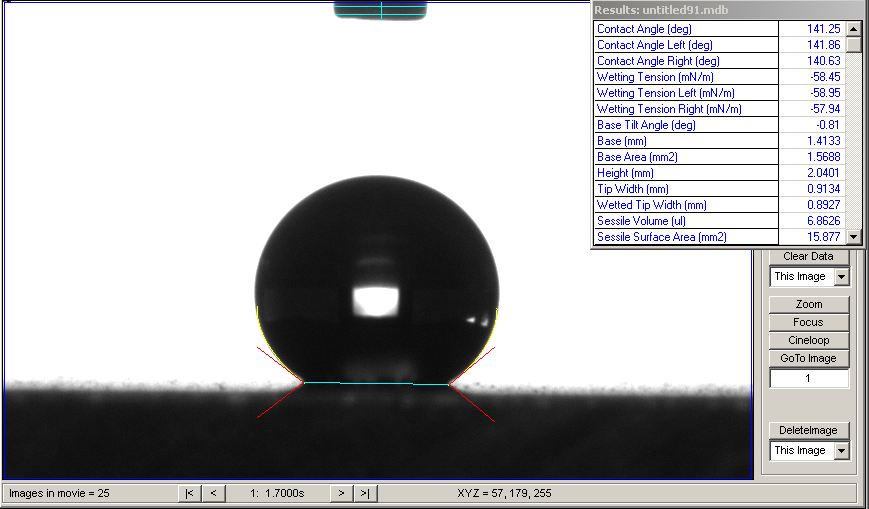


**Figure 15** Validation of the predicted solution 1 (with CA 141.87º at 97.9% desirability) showing the measured contact angle of 141.25 º.


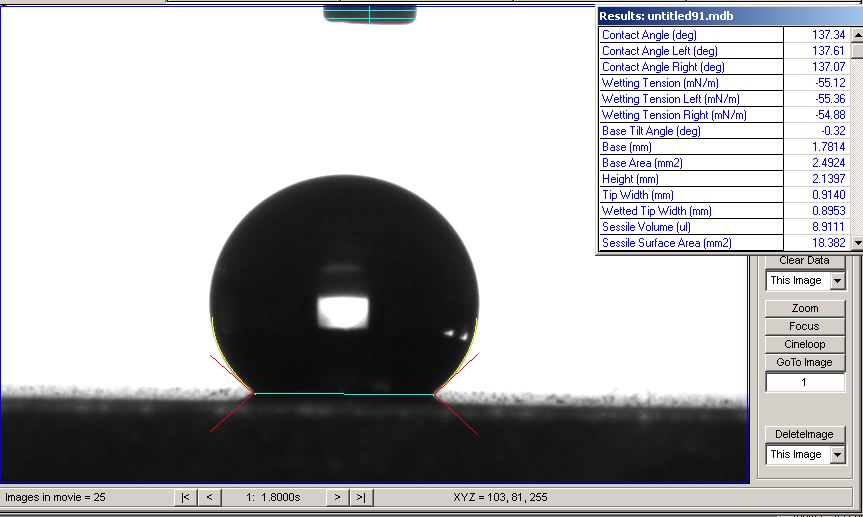


**Figure 16** Validation of the predicted solution 1 (with CA 141.064 º at 97% desirability) showing the measured contact angle of 137.34 º.


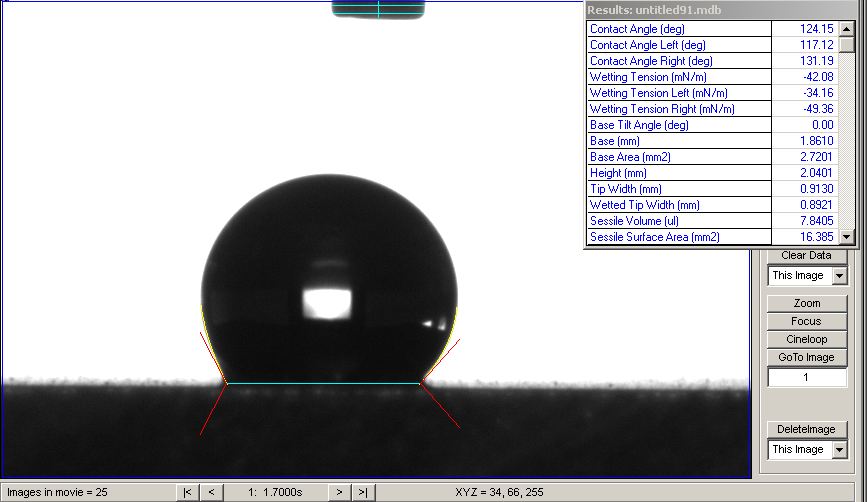


**Figure 17** Validation of the predicted solution 1 (with CA 132.685º at 87.1% desirability ) showing the measured contact angle of 124.15 º.

# Results of XPS analysis data

**Figure 18**. XPS of the aluminium alloy 7075 sample without laser texturing

a. **Day 1 Day 60**

b. **Day 1 Day 60**

**c. Day 1 Day 60**

**d. Day 1 Day 60**

**Figure 19** XPS of the aluminium alloy 7075 with laser texturing XPS done on day 1 and 60

a. sample 1, b. sample 12, c. sample 17 and d. sample 23
